# Impact of type of minimally invasive approach on open conversions across ten common procedures in different specialties

**DOI:** 10.1007/s00464-022-09073-5

**Published:** 2022-02-09

**Authors:** Paresh C. Shah, Alexander de Groot, Robert Cerfolio, William C. Huang, Kathy Huang, Chao Song, Yanli Li, Usha Kreaden, Daniel S. Oh

**Affiliations:** 1 Division of General Surgery, Department of Surgery, Robert I. Grossman School of Medicine, New York University, New York, NY USA; 2 Global Access Value Economics, Intuitive Surgical, Sunnyvale, CA USA; 3 Division of Thoracic Surgery, Department of Cardiothoracic Surgery, Robert I. Grossman School of Medicine, New York University, New York, NY USA; 4 Division of Urologic Oncology, Department of Urology, Robert I. Grossman School of Medicine, New York University, New York, NY USA; 5 Division of Gynecologic Oncology, Department of Obstetrics and Gynecology, Robert I. Grossman School of Medicine, New York University, New York, NY USA; 6 Division of Thoracic Surgery, Department of Surgery, Keck School of Medicine, University of Southern California, 1510 San Pablo St, Los Angeles, CA 90033 USA

**Keywords:** Minimally invasive surgery, Open conversion, Robotic-assisted surgery, General surgery, Gynecological and urological surgery, Thoracic surgery

## Abstract

**Background:**

Conversion rates during minimally invasive surgery are generally examined in the limited scope of a particular procedure. However, for a hospital or payor, the cumulative impact of conversions during commonly performed procedures could have a much larger negative effect than what is appreciated by individual surgeons. The aim of this study is to assess open conversion rates during minimally invasive surgery (MIS) across common procedures using laparoscopic/thoracoscopic (LAP/VATS) and robotic-assisted (RAS) approaches.

**Study design:**

Retrospective cohort study using the Premier Database on patients who underwent common operations (hysterectomy, lobectomy, right colectomy, benign sigmoidectomy, low anterior resection, inguinal and ventral hernia repair, and partial nephrectomy) between January 2013 and September 2015. ICD-9 and CPT codes were used to define procedures, modality, and conversion. Propensity scores were calculated using patient, hospital, and surgeon characteristics. Propensity-score matched analysis was used to compare conversions between LAP/VATS and RAS for each procedure.

**Results:**

A total of 278,520 patients had MIS approaches of the ten operations. Conversion occurred in 5% of patients and was associated with a 1.77 day incremental increase in length of stay and $3441 incremental increase in cost. RAS was associated with a 58.5% lower rate of conversion to open surgery compared to LAP/VATS.

**Conclusion:**

At a health system or payer level, conversion to open is detrimental not just for the patient and surgeon but also puts a significant strain on hospital resources. Use of RAS was associated with less than half of the conversion rate observed for LAP/VATS.

**Supplementary Information:**

The online version contains supplementary material available at 10.1007/s00464-022-09073-5.

The fundamental tenet of minimally invasive surgery (MIS) is to perform an operation through small incisions to decrease tissue trauma, reduce pain, and accelerate recovery without compromising physiologic function or oncologic outcomes. As our technology has improved, we have been able to expand the benefits of MIS to additional modes of value including advanced imaging, new energy tools, among others. Over the last three decades, numerous studies in multiple specialties have demonstrated many benefits of MIS over open surgery, such as lower post operative pain, shorter length of stay, lower blood loss, and often fewer complications; this ultimately translates into improved healthcare quality and reduced resource utilization [1–6]. However, conversion of an MIS operation to an open laparotomy or thoracotomy can result in loss of these benefits, and is detrimental to the patient, surgeon, and hospital [7–9]. While conversions for an individual surgeon may be uncommon, the cumulative effect of conversions during common operations within a hospital or health system could be significant.

Over time, multiple specialties have increasingly adopted robotic-assisted surgery (RAS) as an alternative to laparoscopic/video-assisted thoracoscopic surgery (LAP/VATS), including the fields of urology, gynecology, general surgery, and thoracic surgery. The clinical value of RAS in these fields has been evaluated in their respective specialty silos with specific perioperative outcomes relevant to those procedures [10–15]. However, as hospitals and health systems increasingly invest in multi-specialty robotic programs and in patient-level quality outcomes, there is a need for a more holistic assessment of value of RAS in comparison to the alternative of LAP/VATS. From the perspective of a hospital, health system, or payer, it is of great interest to determine if RAS can add consistent clinical and economic value across the board, not just for one specialty. In this context, conversion rate is an important metric of performance for an MIS program that crosses specialty lines.

The aim of this study was to quantify the impact of conversions on both clinical and financial aspects of a multi-specialty MIS program, taking the perspective of a hospital or health system rather than just an individual surgeon or patient. We further sought to compare the differences in conversion rates between two minimally invasive techniques—RAS and LAP/VATS—across a variety of common procedures typically performed in most hospitals. This study was not designed to assess the specific drivers of conversion (Fig. 1).

## Methods

### Data source

The Premier Healthcare Database (PHD) was used for this study. This database captures more than 103 million visits per year, representing approximately 25% percent of annual US inpatient samples, from over 700 hospitals in the USA [16]. It provides hospital encounter level data, including clinical, billing, and financial data. Patients can be tracked within the same hospital. As this study is an observational study of de-identified patients in a commercially available database without the possibility for re-identification, Institutional Review Board approval was not required.

### Study population

Adult patients (age ≥18 years) who underwent any of the following procedures between January 1, 2013 and September 30, 2015 were included in the study: (1) hysterectomy for benign conditions, (2) hysterectomy for endometrial cancer, (3) sigmoidectomy for diverticular disease, (4) low anterior resection for rectal cancer, (5) right colectomy for benign conditions, (6) right colectomy for malignant conditions, (7) inguinal hernia repair, (8) ventral hernia repair, (9) lobectomy for primary lung cancer, and (10) partial nephrectomy for kidney cancer. These ten procedures represent a diverse group of high-volume surgical procedures in which both RAS and LAP/VATS approaches are commonly used and represent the specialties with the highest utilization of MIS. Each procedure had to have at least 10% adoption for either RAS or LAP/VATS.

International Classification of Diseases, Ninth Revision [ICD-9], Current Procedural Terminology [CPT] codes and hospital billing data were used to define the eligible cases and surgical modality [Supplemental Table 1]. If an individual had multiple of the same procedure, only the individual’s first qualified procedure was included in the study. Additionally, individuals were excluded if the operating room time or total cost for their initial hospitalization was not greater than zero, if any of the patient or provider/hospital characteristics needed for the analysis were missing, or if specific exclusion criteria for surgical indications were met [Supplemental Table 1].

### Study variables

The primary outcome of this study was the conversion to laparotomy/thoracotomy from RAS or LAP/VATS based on ICD-9 codes (V64.4X). Conversion from RAS to LAP/VATS or vice versa was not included as this level of detail could not be reliably assessed in Premier. Secondary outcomes included the length of stay (LOS), 30-day readmission rate, and total cost for initial hospitalization/visit and during the first 30 days following surgery compared between the MIS and converted-to-open cohorts.

Patient characteristics analyzed included age, race, patient admission type (inpatient or outpatient), insurance type (Medicare, Medicaid, commercial, or self-pay/others), Charlson Comorbidity Index (CCI), body mass index (BMI) category, and year of surgery. Surgeon characteristics included physician specialty and surgeon volume (calculated as the number of times the performing surgeon conducted the procedure in the one year prior to the date of surgery at that hospital). Individual surgeon volume for each procedure was used to generate low-volume, medium-volume, and high-volume categories. The cutoffs for volume were set as terciles based on the range of individual annual surgeon volumes for each procedure regardless of modality. Hospital characteristics included location (rural or urban), hospital type (community or teaching), US region (Midwest, Northeast, South, or West), and bed number (<200, 200–499, 500+).

Length of stay (LOS) was captured in the database Premier from admission to discharge, including actual discharge dates. LOS-related analyses were limited to those procedures that are routinely performed in the inpatient setting, including LAR, lobectomy, partial nephrectomy, right colectomy and sigmoidectomy. A patient readmission within 30 days was defined by the patient being readmitted to the same hospital where surgery had been performed within 30 days after discharge. Readmission to another hospital is not tracked in Premier. The perioperative 30-day period was defined as from admission day through 30 days after discharge. Cost data were captured through actual hospital cost reported in Premier, including fixed (overhead) and variable (direct) costs. The costs were adjusted for inflation to 2015 US dollar using the historical Consumer Price Index for All Urban Consumers. Total cost during the initial visit and perioperative 30-day periods were calculated.

### Statistical analysis

Descriptive analyses of patient, physician and hospital characteristics were performed for different surgical approaches. Frequencies and percentages for categorical variables were reported and X^2^ tests or Fisher exact tests were used to examine the difference. Medians, and interquartile ranges for continuous variables were reported and Wilcoxon rank sum test was used to examine the difference between groups.

In order to minimize selection bias, we conducted a propensity score matched (PSM) analysis to compare conversion rates in RAS and LAP/VATS procedures. The propensity of receiving RAS was generated for each visit through multivariable logistic regression adjusted for patient, physician and provider characteristics previously listed. For most procedures, 1 to 1 greedy matching without replacement was used to generate the matched study samples [17]. Given that the numbers of patients who underwent LAP are relatively small for hysterectomy for endometrial cancer and partial nephrectomy, 1 to 3 greedy matching without replacement was used instead (1 LAP/VATS to 3 RAS). The adequacy of matching was evaluated using standardized difference with a threshold of <0.1 indicating a negligible difference [18].

Within matched pair cohorts, conversion rates between RAS and LAP/VATS cohorts were calculated. X^2^ tests or Fisher exact tests were used to examine the difference of conversion rates among the unmatched and matched cohorts. If distributions of some covariates remained unbalanced after PSM, logistic regression was used to additionally adjust for those unbalanced covariates. Conversion rates for each procedure were weighted by procedure volume during study periods and summed up to total conversion rates. Absolute difference (Conversion rate of RAS-conversion rate of LAP) and relative differences (Conversion rate of RAS-conversion rate of LAP)/conversion rate of LAP) were calculated to evaluate the difference between the two groups.

To better understand the consequence of conversion, multivariate logistic regression was conducted to assess 30-day readmission rates and gamma regression was conducted to assess length of stay, total cost for index hospitalization and the perioperative 30-day window between MIS and converted cases, adjusting for all patient characteristics and hospital/provider characteristics listed above. Adjusted risk differences for length of stay and total cost and adjusted risk ratio of 30-day readmission were reported. The overall risk ratio and risk difference across all the procedures were calculated as the weighted sum of the risk ratio and risk difference for each procedure by their surgical volume during the surgical period.

All tests were 2-sided. A *p* value less than 0.05 was considered statistically significant. All analyses were conducted using SAS software version 9.4 (SAS Institute Inc).

## Results

**Fig. 1:**
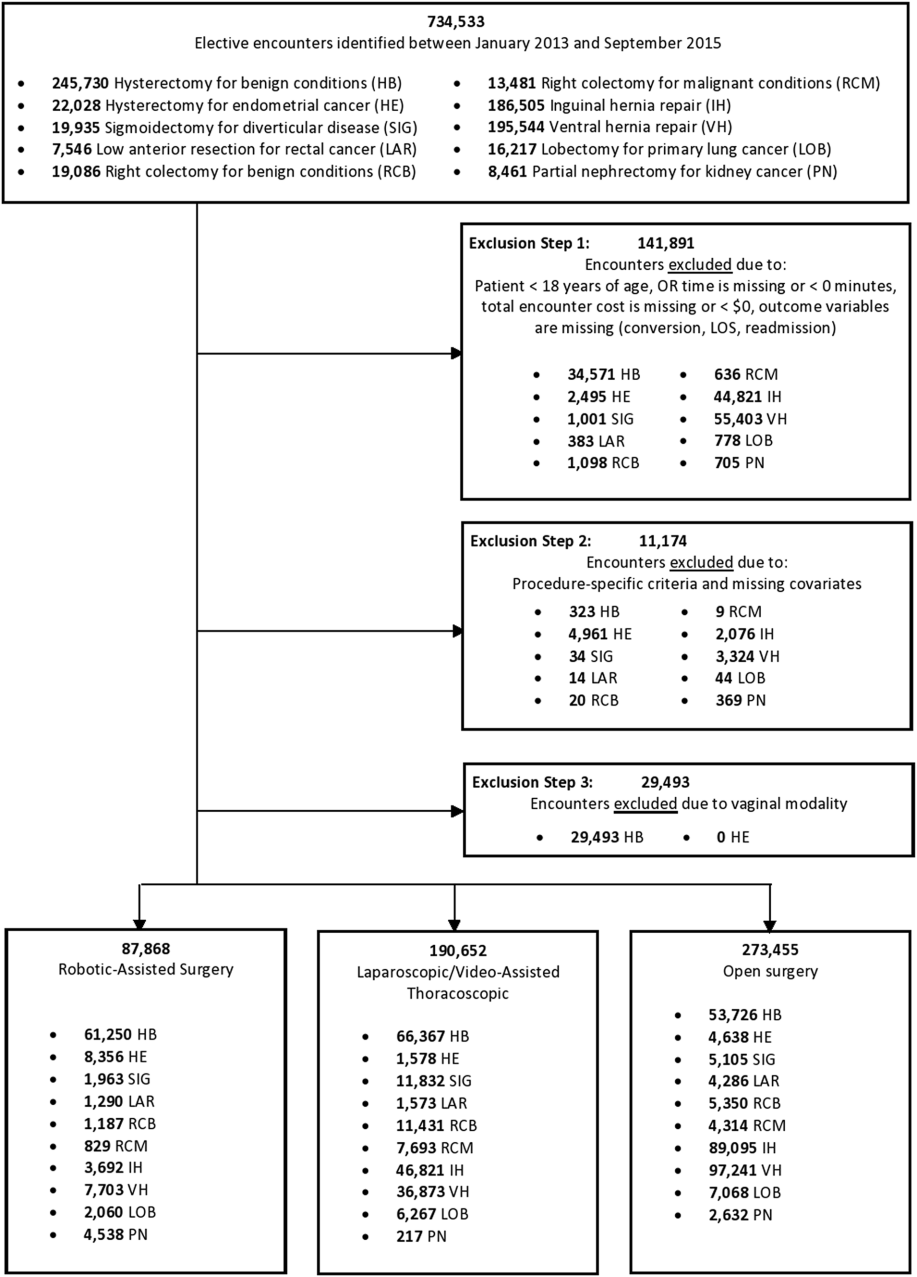
Study flowchart

A total of 734,533 patients underwent the selected ten procedures during the study period in the Premier database. Of these, 551,975 patients met selection criteria (Figure 1), which included open and MIS approaches, and were included in the study (Table 1): 181,343 (33%) patients had hysterectomy for benign conditions, 14,572 (3%) had hysterectomy for endometrial cancer, 18,900 (3%) had sigmoidectomy for diverticular disease, 7149 (1%) had low anterior resection for rectal cancer, 17,968 (3%) had right colectomy for benign conditions, 12,836 (2%) had right colectomy for malignant conditions, 139,608 (25%) had inguinal hernia repair, 136,817 (25%) had ventral hernia repair, 15,395 (3%) had lobectomy for primary lung cancer, and 7387 (1%) had partial nephrectomy for kidney cancer. The adoption of minimally invasive surgery (RAS and lap/VATS) varied across procedures, from as high as 73% for sigmoidectomy to as low as 29% for ventral hernia repair. A total of 13,969 conversions to open were observed out of 278,520 minimally invasive operations during the study period, with an overall conversion rate of 5%, ranging from a low of 1% in inguinal hernia repair to a high of 24% in LAR procedures.

**Table 1:** Utilization of open, laparoscopic/thoracoscopic (LAP/VATS), and robotic-assisted surgery (RAS) for each procedure, with the total minimally invasive surgery (MIS) adoption rate and rate of conversion to open

Procedure	Total patients	Open	MIS	Conversion to open from MIS
		N (%)	N (%)	N (% of MIS)
*Benign procedures*				
Sigmoidectomy for diverticular disease	18,900	5105 (27%)	13,795 (73%)	1709 (12%)
Hysterectomy for benign conditions	181,343	53,726 (30%)	127,617 (70%)	5182 (4%)
Right colectomy for benign conditions	17,968	5350 (30%)	12,618 (70%)	1347 (11%)
Ventral hernia repair	136,817	97,241 (71%)	39,576 (29%)	1870 (5%)
Inguinal hernia repair	139,608	89,095 (64%)	50,513 (36%)	729 (1%)
*Malignant procedures*				
Low anterior resection for rectal cancer	7149	4286 (60%)	2863 (40%)	701 (24%)
Hysterectomy for endometrial cancer	14,572	4638 (32%)	9934 (68%)	598 (6%)
Partial nephrectomy for kidney cancer	7387	2632 (36%)	4755 (64%)	125 (3%)
Right colectomy for malignant conditions	12,836	4314 (34%)	8522 (66%)	914 (11%)
Lobectomy for primary lung cancer	15,395	7068 (46%)	8327 (54%)	794 (10%)
Total	551,975	273,500 (50%)	278,520 (50%)	13,969 (5%)

### MIS cases vs. converted cases

**Fig. 2:**
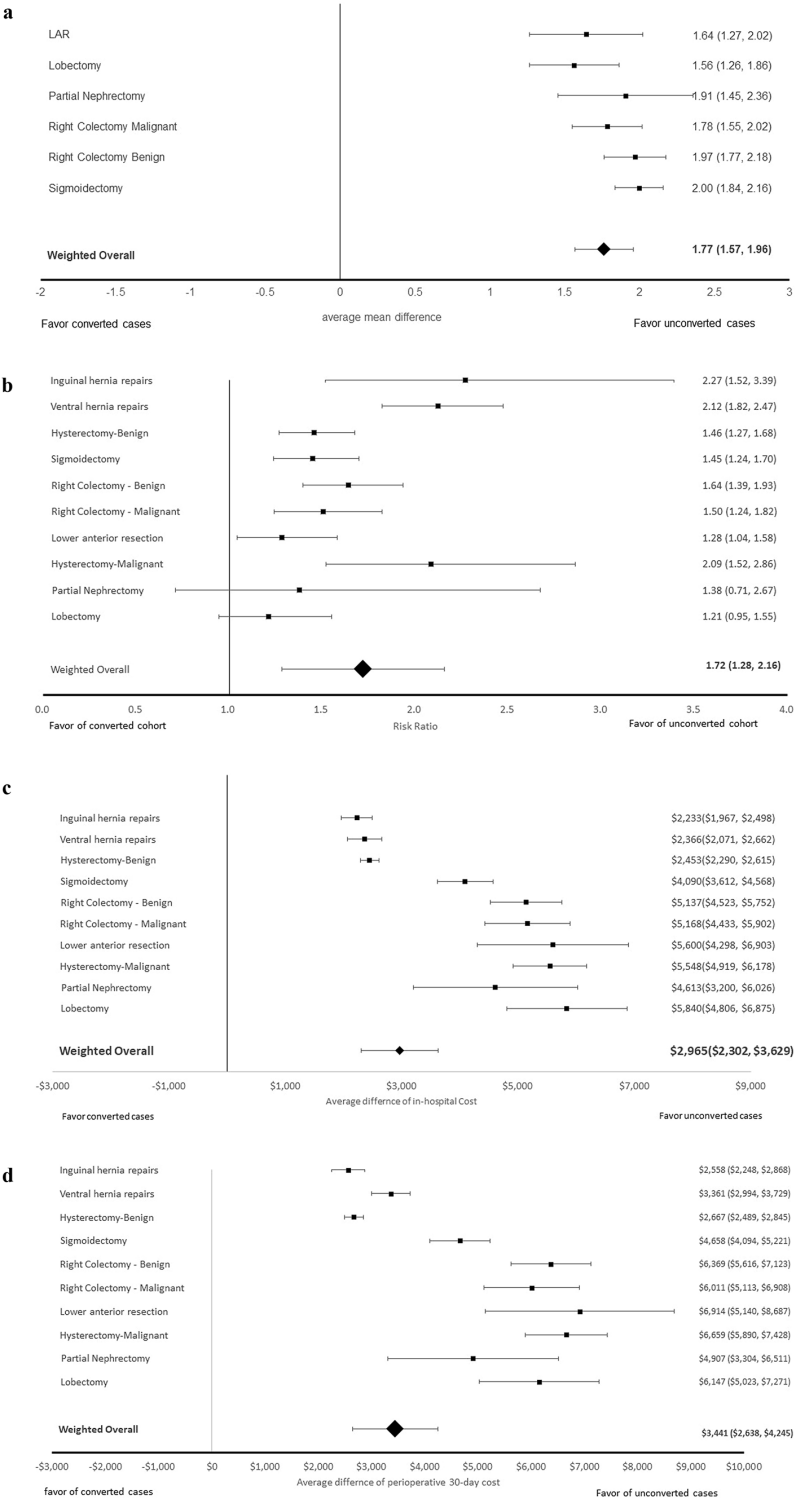
(**A**) Impact of conversion on length of stay compared with MIS cases (excludes outpatient procedure types). The point estimate and 95% confidence interval are shown. (**B**) Impact of conversion on postoperative 30-day readmission compared with MIS cases. The point estimate and 95% confidence interval are shown. (**C**) Impact of conversion on in-hospital total cost comparing with MIS cases. The point estimate and 95% confidence interval are shown. (**D**) Impact of conversion on perioperative 30-day total cost comparing with MIS cases. The point estimate and 95% confidence interval are shown

Compared to MIS patients without conversion, converted-to-open patients were associated with longer length of stay across all inpatient procedures (overall additional 1.77 days, 95% CI [1.57, 1.96]). The impact of conversion on incremental additional LOS ranged from 1.56 to 2.00 days across different procedures (Fig. 2A).

There was a significantly increased risk of readmission during the first 30 days following surgery for converted cases for all procedures combined (risk ratio 1.72, 95% CI [1.28, 2.16]). At an individual procedure level, the risk of readmission for MIS cases was significantly lower than for converted cases in all ten procedures except for lobectomy and partial nephrectomy (Fig. 2B).

The perioperative 30-day total cost of converted cases were significantly higher compared with MIS cases for all procedures (additional incremental cost of $3441, 95% CI [$2638, $4245]). The most pronounced difference of total cost between converted cases and MIS cases was observed for LAR (additional cost of $6914, 95% CI [$5149, $8587]), while the least difference of total cost was observed for inguinal hernia repair (additional cost of $2558, 95% CI [$2448, $2868]). Similar results were observed for total cost during the hospitalization (Fig. 2C, D).

### Converted cases vs. primary open cases

When compared to patients who had primary open surgery, converted MIS cases had longer LOS for several inpatient operations: LAR (additional 0.96 day LOS, 95% CI [0.62, 1.31]), partial nephrectomy (additional 0.61 day, 95% CI [0.17, 1.04]) and malignant right colectomy (additional 0.37 days, 95% CI [0.10, 0.63]) (Supplemental Fig. 1A).

The risk of 30-day readmission rate was significantly higher for converted MIS cases compared to primary open cases for three procedures: inguinal hernia repair (risk ratio 2.05, 95% CI [1.38, 3.05]), ventral hernia repair (risk ratio 1.38, 95% CI [1.20, 2.58]), and benign hysterectomy (risk ratio 1.50, 95% CI [1.32, 1.70] (Supplemental Fig. 1B).

Total cost during hospitalization and within the first 30 days following surgery was significantly higher for converted MIS cases compared to primary open cases for all ten procedure types (Supplemental Fig. 1C and 1D).

### RAS vs LAP/VATS cases

Within the MIS cohort, the utilization rate of RAS varied among the different procedures. The highest proportion of RAS was 57% in hysterectomy for endometrial cancer and the lowest was 2% in ventral hernia repairs. To compare the differences in conversion between the LAP/VATS and RAS cohorts, PSM was performed (characteristics before and after PSM are shown in Supplemental Table 2. 1–10). After matching, the groups were well balanced (standardized difference <0.1) for all covariates except for region and surgeon specialty for lobectomy and surgeon volume for hysterectomy for endometrial cancer; these were further adjusted by logistic regression.

Among the matched cohorts, conversion rates for RAS were significantly lower than for LAP/VATS across all procedures (Table 2). The volume-weighted conversion rate for RAS was 2.8%, compared to 6.5% conversion rate for LAP. The relative conversion reduction of RAS compared to LAP/VATS varied from 26.8% in benign right colectomy to 83.3% in partial nephrectomy. The total relative reduction in conversions for all procedures with RAS was 58.5% compared to LAP/VATS.

**Table 2:** Conversion rates among patients who underwent laparoscopic/thoracoscopic (LAP/VATS) and robotic-assisted surgery (RAS)

	LAP/VAS		RAS		*P*-value	Absolute diff	Relative diff
	N/N after PSM	% converted	N/N after PSM	% converted			
*Benign procedures*							
Sigmoidectomy for diverticular disease	11,832/1963	11.30%	1963/1963	8.30%	0.001	−3.0%	−26.5%
Benign hysterectomy	66,369/47,673	5.60%	61,253/47,673	1.90%	<0.0001	−3.7%	−66.1%
Benign right colectomy	11,431/1187	10.70%	1187/1187	7.80%	0.02	−2.9%	−27.1%
Ventral hernia repair	37,368/2703	6.20%	2744/2703	3.60%	<0.0001	−2.6%	−41.9%
Inguinal hernia repair	46,990/3692	2.10%	3720/3692	0.80%	<0.0001	−1.3%	−61.9%
*Malignant procedures*							
Low anterior resection for rectal cancer	1573/940	31.1%	1290/940	11.40%	<0.0001	−19.7%	−63.3%
Hysterectomy for endometrial cancer	1578/987	16.4%	8357/2961	4.40%	<0.0001^a^	−12.0%	−73.1%
Partial nephrectomy for kidney cancer	217/210	13.3%	4540/630	3.00%	<0.0001^b^	−10.3%	−77.5%
Malignant right colectomy	7693/829	11.2%	829/829	6.90%	0.002	−4.3%	−38.5%
Lobectomy for primary lung cancer	6268/1966	8.6%	2060/1966	6.30%	0.005^c^	−2.3%	−26.7%
Total	191,319/62,150	6.4%	87,943/64,544	2.7%	<0.0001	−3.8%	−58.5%

^a^Standardized difference of physician volume after propensity score matching >0.10. *P*-values are further adjusted physician volume

^b^Standardized difference of provider region after propensity score matching >0.10. *P*-values are further adjusted provider region

^c^Standardized difference of physician specialty after propensity score matching >0.10. *P*-values are further adjusted physician specialty

## Discussion

To our knowledge, this is the first publication to analyze the impact of conversion across multiple, commonly performed operations across different specialties. Our data indicate that the conversion rate to laparotomy or thoracotomy varies considerably among different procedures, but there is consistent, detrimental impact on length of stay, readmissions, and 30-day costs. In the current setting of healthcare resource constraints, the cumulative effect of these conversions is significant at the health system level. Reducing conversions during MIS has a meaningful impact on patient outcomes and value. While the reasons for conversion could be heterogeneous and dependent on several factors, we observed a consistent and significant decrease in conversion associated with RAS compared to LAP/VATS across all procedures studied. This manifested with a 58.5% relative reduction in conversion events. While many factors contribute to a conversion that are difficult to control for, the use of a robotic-assisted approach appears to correlate with a lower rate of conversion and thus better likelihood of completing the operation in a minimally invasive manner.

One consistent observation in these data is the finding of reduced conversion associated with RAS across all procedures and specialties. The range of these operations is diverse, from extirpative procedures such as hysterectomy and lobectomy, to those that require reconstruction such as colectomy, and to procedures that restore normal anatomy and function such as hernia repair. It is acknowledged that the primary endpoint of an operation is not to avoid conversion, but to accomplish the task at hand—remove the diseased organ, reconstruct physiology, and/or restore normal anatomy. Reducing conversions does not justify worse clinical outcomes for the patient. Data from previous publications in different specialties indicate that for clinical outcomes, RAS is associated with similar—or in some instances, better—results than their respective LAP/VATS alternative approaches [10, 14, 19–24]. This body of evidence strongly indicates that RAS has potential advantages that extend beyond technical avoidance of conversion.

It is generally accepted and agreed that at an individual case level, conversion should not be regarded as a complication or a failure. However, in the aggregate, conversion rates are also generally accepted as a measure of quality. Many factors at the surgeon and patient level can impact conversion rates, beginning with surgeon skill, experience and patient selection. The reason for a conversion can broadly be categorized into two causes—emergency conversions due to a catastrophic event such as major bleeding and elective conversions due to the inability to make progress during the operation. A notable limitation of this analysis is that due to the constraints in the Premier database we are unable to discern the cause of conversion. There is emerging data to suggest that RAS is associated most often with a reduction in the elective conversions, and emergency conversions remain similar [25]. The reason for the significant decrease in conversion with RAS could be a reflection of some technological advantages of the robotic platform compared to a manual MIS approach. These technological advantages include better vision with stable 3D high definition video, articulating instruments that mimic the wristed ergonomics of open surgery, tremor filtration, motion scaling, and the autonomy to control all visualization and instrument arms. This study cannot determine causality, but these technological differences could contribute to the observed differences.

There are many limitations to this study that are inherent in the retrospective analysis of use of a large aggregate database. There are many potentially confounding variables such as surgeon and/or patient characteristics that we cannot account for. It is possible that surgeons who adopt RAS are early adopters who tend to have higher volume or more experience than those who utilize LAP/VATS techniques, resulting in better clinical outcomes. It is also possible that hospitals that have wider adoption of RAS technology have more financial resources, resulting in better patient care. With PSM, we sought to minimize these influences but we acknowledge that in a retrospective, real-world analysis, these statistical methods cannot fully mitigate this. On the patient side, cancer-related factors such as tumor size or degree of invasion could influence conversions rates. While such details are not captured in the Premier database, it can be reasonably assumed that surgeons using either MIS platform would select patients with similar tumor characteristics, unlike for open cases. Another concern in this analysis is whether conversion includes exploratory laparoscopy/thoracoscopy performed for a cancer operation with the intent to proceed in an open operation if no metastases are present. We excluded these types of cases as they have separate codes. Moreover, the consistent decrease in conversion was also observed in benign procedures, not just malignant ones.

Other patient factors could influence conversion such as prior surgical history and obesity. However, similar to tumor characteristics, one would not reasonably expect selection bias toward RAS instead of LAP/VATS on this basis alone. It is possible that robotic technology facilitates the operation in obese patients compared to manual MIS approaches due to the absence of physical strain on the surgeon and could result in decreased conversions. For an obese patient, positioning such as in reverse Trendelenburg for a pelvic operation may also influence the inability to tolerate an MIS operation and would be expected to increase conversions. However, this would presumably affect all MIS (RAS and LAP/VATS) procedures equally.

A notable limitation of this study is that the data are high-level and granular details such as the reason for conversion are not provided in the Premier database. Dependency on ICD-9 or CPT codes could result in inconsistent outcomes. Moreover, Premier captures a fraction of hospital procedures in the USA and is not randomly sampled. Operative reports are not available for review, and surgeons cannot be interviewed to determine the exact reason for conversion. Moreover, Premier data do not allow tracking of patients or surgeons across different hospitals, so readmission and surgeon volume are limited to the site of the primary operation. As a retrospective study, selection bias is a possibility between the two MIS techniques, although adjustment for various patient, surgeon, and hospital characteristics was performed to minimize this issue. For procedures that have both a significant inpatient as well as outpatient case mix (such as ventral hernia), there may be patient disease-specific factors that influence the decision to be admitted or not—for this reason, these procedures were not included in the LOS analysis. Moreover, operations that can be done either as outpatient or inpatient may have selection bias, in which converted patients are preferentially admitted as a result of the conversion itself. We have attempted to mitigate any selection bias by including inpatient/outpatient status as a variable in propensity score matching and multivariate regression. Lastly, surgeon training and access to the platform represent additional sources of potential bias that we can neither account nor control for in this analysis. It also apparent that utilization of RAS is at different levels of maturity among the various procedures and specialties, which could influence overall outcomes. Certain specialties such as gynecology and urology have already achieved a majority of robotically trained and proficient surgeons, whereas the penetration in general and colon rectal surgery is still very modest, representing a minority of surgeons in that field.

## Conclusion

The results of this real-world body of evidence indicates that conversion to open from an MIS approach results in detrimental outcomes with significant resource utilization. From the standpoint of population health or a hospital system, these high-level data indicate that the cumulative effect of conversions can be a significant burden, and that reduction of conversions has major benefits and leads to increased value for the patient, the hospital, and society at large. The use of RAS is associated with a significant decrease in the conversion rate for all ten operations studied, and a multidisciplinary robotic program encompassing several specialties could result in significantly decreased conversion rates with an improved ability to deliver successful MIS to its patients.

## Disclosures

All the authors have no received payment or service from a third party for the submitted work. Alexander de Groot, Dr. Yanli Li, Chao Song, Usha Kreaden reported full-time employment from Intuitive Surgical outside the submitted works. Dr. Daniel Oh reported part-time employment as a medical advisor from Intuitive Surgical outside the submitted works. Dr. Kathy Huang reported consultancy from Intuitive surgical and Johnson & Johnson outside the submitted works. Dr. William Huang and Dr. Robert Cerfolio reported consultancy from Intuitive surgical outside the submitted works. Dr. Paresh Shah reported board membership of CareCentra, consultancy and payment for lecture speaker and development of educational presentations from Intuitive Surgical, outside the submitted work.

## Supplementary Information

Below is the link to the electronic supplementary material.

Supplementary file1 (JPG 51 KB)

Supplementary file2 (JPG 63 KB)

Supplementary file3 (JPG 68 KB)

Supplementary file4 (JPG 68 KB)

Supplementary file5 (DOCX 22 KB)

Supplementary file6 (DOCX 101 KB)

## Abbreviations

MIS: Minimally invasive surgery
RAS: Robotic-assisted surgery
LAP: Laparoscopic surgery
VATS: Video-assisted thoracoscopic surgery
PHD: Premier Healthcare Database
CCI: Charlson Comorbidity Index
BMI: Body mass index
