# Supplementary figures and images for: Impact of type of minimally invasive approach on open conversions across ten common procedures in different specialties
Source: Surg Endosc. 2022 Feb 9;36(8):6067–75. doi: 10.1007/s00464-022-09073-5 (PMC9283176; doi:10.1007/s00464-022-09073-5)

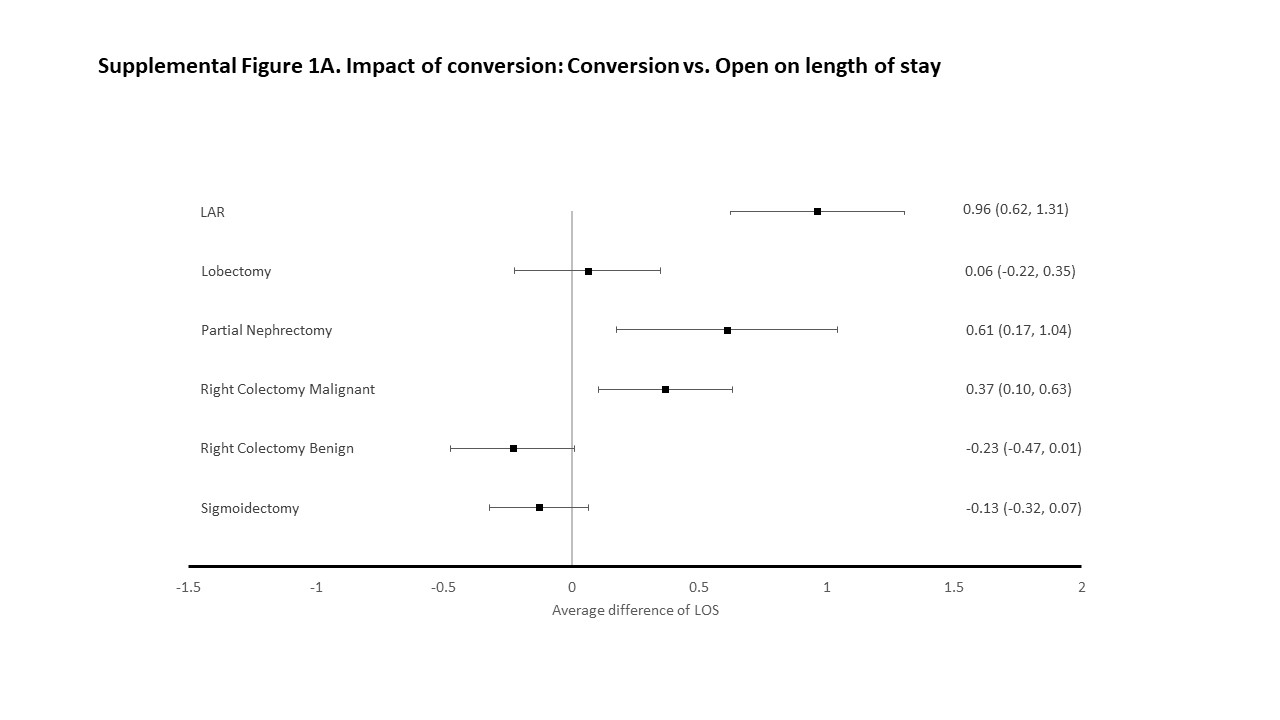

Supplement: Supplementary file 1 — Supplementary file1 (JPG 51 KB) [file 464_2022_9073_MOESM1_ESM.jpg]

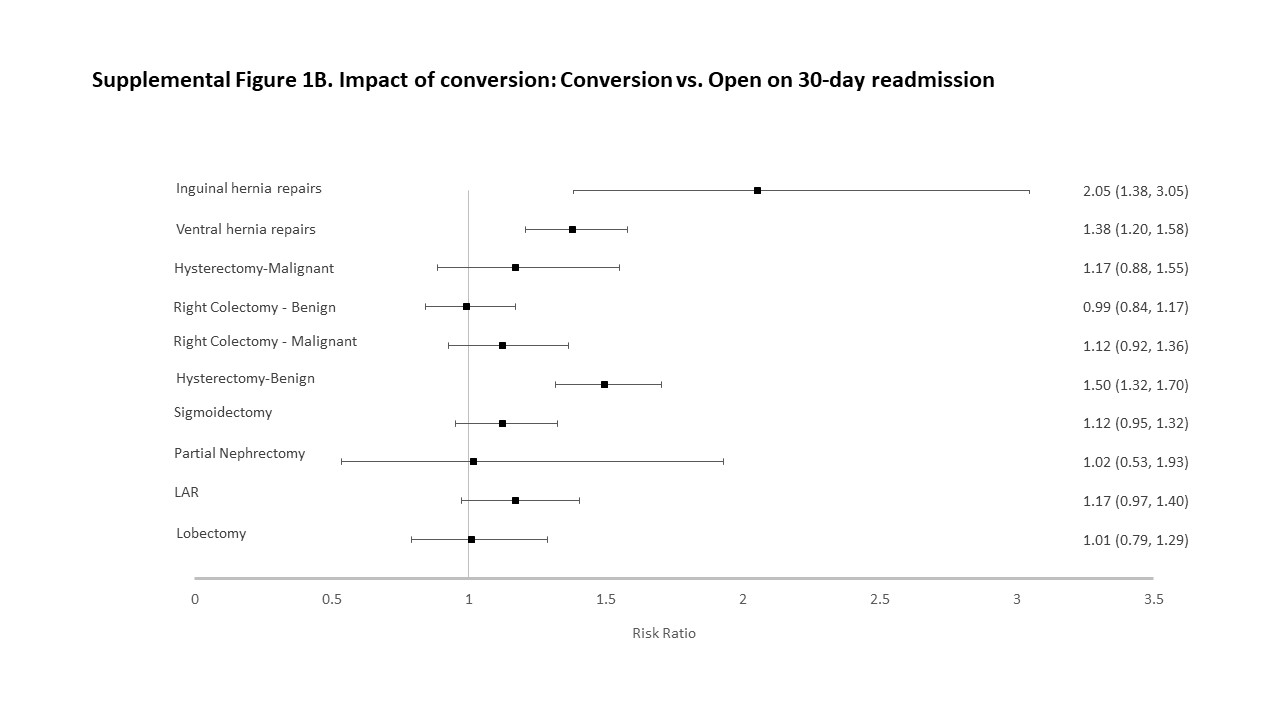

Supplement: Supplementary file 2 — Supplementary file2 (JPG 63 KB) [file 464_2022_9073_MOESM2_ESM.jpg]

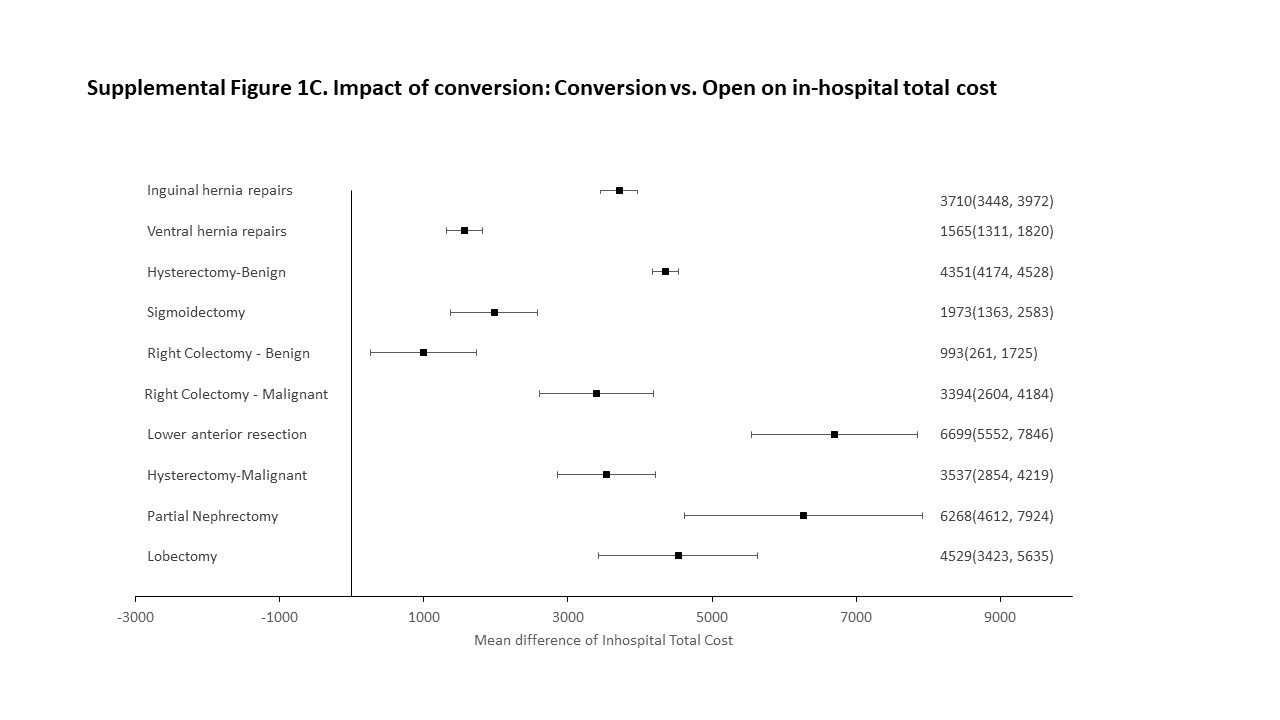

Supplement: Supplementary file 3 — Supplementary file3 (JPG 68 KB) [file 464_2022_9073_MOESM3_ESM.jpg]

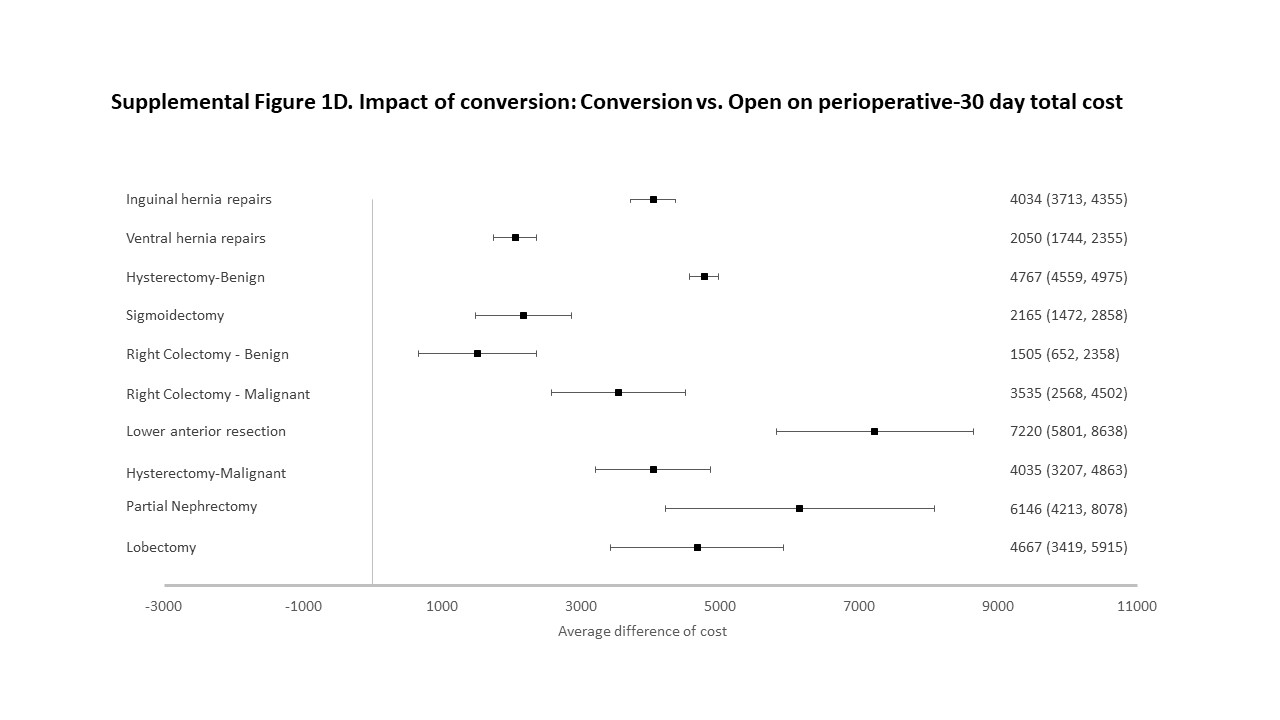

Supplement: Supplementary file 4 — Supplementary file4 (JPG 68 KB) [file 464_2022_9073_MOESM4_ESM.jpg]
