# Supplementary material for: Impact of type of minimally invasive approach on open conversions across ten common procedures in different specialties
Source: Surg Endosc. 2022 Feb 9;36(8):6067–75. doi: 10.1007/s00464-022-09073-5 (PMC9283176; doi:10.1007/s00464-022-09073-5)
Supplement: Supplementary file 5 — Supplementary file5 (DOCX 22 KB) [file 464_2022_9073_MOESM5_ESM.docx]

**Supplemental Table 1. Case and modality definitions**

| **Procedure** | **Inclusion Codes** | **Exclusion Codes** | **Modality Definitions** |
| --- | --- | --- | --- |
| Lobectomy for Primary Lung Cancer | Lobectomy Procedure   - ICD-9-Proc: 32.4X (P)   AND  Primary Lung Cancer   - ICD-9-Dx: 162.X | None | Robotic:   - CPT/HCPCS: S2900 - ICD-9-Proc: 17.4X - Robotic billing record   VATS:   - CPT/HCPCS: 32663, 32668, 32670 - ICD-9-Proc: 32.41 - ICD-9-Dx: V64.41, V64.42 - Not robotic   Open:   - If none of the above |
| Hysterectomy for Benign Conditions | Hysterectomy Procedure:   - CPT: 58150, 58152, 58180, 58200, 58260, 58262, 58263, 58267, 58270, 58275, 58280, 58290, 58291-58294, 58541-58544, 58550, 58552-58554, 58570-58573 - ICD-9-Proc: 68.3X (P), 68.4X (P), 68.5X (P), 68.9 (P)   AND  **Without** Gynecological Malignancy:   - ICD-9-Dx: 179, 180.X, 181, 182.X, 183.X, 184.X, 198.6, 198.82, 233.1-233.39, 236.0-236.3, 795.06 | Radical Hysterectomy:   - ICD-9-Proc: 68.6X, 68.7X - CPT/HCPCS: 58210, 58240, 58285, 58548   Pelvic or Lower Abdominal Trauma:   - ICD-9-Dx: 867.4-867.9, 868.00, 868.03-868.09, 868.10, 868.13-868.19, 869.X, 879.6-879.9, 906.0-906.2, 947.4   Pregnancy, Childbirth, or Location in Puerperium:   - Major Diagnostic Category: 14 | Robotic:   - CPT/HCPCS: S2900 - ICD-9-Proc: 17.4X - Robotic billing record   Laparoscopic:   - CPT/HCPCS: 58541-58546, 58548, 58550, 58552-58554, 58570-58573, 58578 - ICD-9-Proc: 68.31, 68.41, 68.51, 68.61, 68.71 - ICD-9-Dx: V64.41 - Not robotic   Vaginal:   - ICD-9-Proc: 68.59, 68.79 - CPT/HCPCS: 58260, 58262, 58263, 58267, 58270, 58275, 58280, 58285, 58290-58294 - Not robotic or laparoscopic   Open:   - If none of the above |
| Hysterectomy for Endometrial Cancer | Hysterectomy Procedure:   - CPT/HCPCS: 58150, 58152, 58180, 58200, 58210, 58240, 58260, 58262, 58263, 58267, 58270, 58275, 58280, 58285, 58290-58294, 58541-58544, 58548, 58550, 58552-58554, 58570-58573 - ICD-9-Proc: 68.3X (P), 68.4X (P), 68.5X (P), 68.6X (P), 68.7X (P), 68.9 (P)   AND  Endometrial Cancer:   - ICD-9-Dx: 182.X | Radical Hysterectomy:   - ICD-9-Proc: 68.6X, 68.7X   Subtotal Hysterectomy:   - ICD-9-Proc: 68.3X, 68.5X, 68.9 | Robotic:   - CPT/HCPCS: S2900 - ICD-9-Proc: 17.4X - Robotic billing record   Laparoscopic:   - CPT/HCPCS: 58541-58546, 58548, 58550, 58552-58554, 58570-58573, 58578 - ICD-9-Proc: 68.31, 68.41, 68.51, 68.61, 68.71 - ICD-9-Dx: V64.41 - Not robotic   Vaginal:   - ICD-9-Proc: 68.59, 68.79 - CPT/HCPCS: 58260, 58262, 58263, 58267, 58270, 58275, 58280, 58285, 58290-58294 - Not robotic or laparoscopic   Open:   - If none of the above |
| Right Colectomy for Benign Conditions | Right Colectomy Procedure:   - ICD-9-Proc: 17.33 (P), 45.73 (P)   AND  **Without** Right Colon Cancer:  ICD-9-Dx: 153.0, 153.4, 153.6 | None | Robotic:   - CPT/HCPCS: S2900 - ICD-9-Proc: 17.4X - Robotic billing record   Laparoscopic:   - CPT/HCPCS: 44204-44206, 44227, 44238 - ICD-9-Proc: 17.31-17.33, 17.39, 45.81, 54.21, 54.51 - ICD-9-Dx: V64.41 - Hand-assist billing record - Not robotic   Open:   - If none of the above |
| Right Colectomy for Malignant Conditions | Right Colectomy Procedure:   - ICD-9-Proc: 17.33 (P), 45.73 (P)   AND  Right Colon Cancer:   - ICD-9-Dx: 153.0, 153.4, 153.6 | None | Robotic:   - CPT/HCPCS: S2900 - ICD-9-Proc: 17.4X - Robotic billing record   Laparoscopic:   - CPT/HCPCS: 44204-44206, 44227, 44238 - ICD-9-Proc: 17.31-17.33, 17.39, 45.81, 54.21, 54.51 - ICD-9-Dx: V64.41 - Hand-assist billing record - Not robotic   Open:   - If none of the above |
| Sigmoidectomy for Diverticular Disease | Sigmoidectomy Procedure:   - ICD-9-Proc: 17.36 (P), 45.76 (P   AND  Diverticulitis:  ICD-9-Dx: 562.1X | Colon or Rectal Malignancy:   - ICD-9-Dx: 153.X, 154.X, 195.2, 195.3, 197.5, 209.14-209.17, 230.3 | Robotic:   - CPT/HCPCS: S2900 - ICD-9-Proc: 17.4X - Robotic billing record   Laparoscopic:   - CPT/HCPCS: 44204, 44213 - ICD-9-Proc: 17.35, 17.36, 54.21, 54.51, 59.03, 59.12 - ICD-9-Dx: V64.41 - Hand-assist billing record - Not robotic   Open:   - If none of the above |
| Low Anterior Resection for Rectal Cancer | LAR Procedure:   - ICD-9-Proc: 48.62 (P), 48.63 (P)   AND  Rectal Cancer:   - ICD-9-Dx: 154.X, 196.2, 197.5, 209.17 | None | Robotic:   - CPT/HCPCS: S2900 - ICD-9-Proc: 17.4X - Robotic billing record   Laparoscopic:   - CPT/HCPCS: 44207, 44208, 44213, 45395, 45397 - ICD-9-Proc: 48.42, 48.51, 54.21, 54.51 - ICD-9-Dx: V64.41 - Hand-assist billing record - Not robotic   Open:   - If none of the above |
| Inguinal Hernia Repair | Inguinal Hernia Repair Procedure:   - CPT/HCPCS: 49505 (O), 49507 (O), 49520 (O), 49521 (O), 49525 (O), 49650 (O), 49651 (O) - ICD-9-Proc: 17.1X (P), 17.2X (P), 53.0X (P), 53.1X (P) | None | Robotic:   - CPT/HCPCS: S2900 - ICD-9-Proc: 17.4X - Robotic billing record   Laparoscopic:   - CPT/HCPCS: 49650, 49651, 49659 - ICD-9-Proc: 17.1X, 17.2X, 54.21, 54.51, 59.03, 59.12 - ICD-9-Dx: V64.41 - Not robotic   Open:   - If none of the above |
| Ventral Hernia Repair | Ventral Hernia Repair:   - CPT/HCPCS: 49560 (O), 49561 (O), 49565 (O), 49566 (O), 49570 (O), 49572 (O), 49585 (O), 49587 (O), 49590 (O), 49652-49657 (O) - ICD-9-Proc: 53.4X (P), 53.5X (P), 53.6X (P) | None | Robotic:   - CPT/HCPCS: S2900 - ICD-9-Proc: 17.4X - Robotic billing record   Laparoscopic:   - CPT/HCPCS: 49652-49657 - ICD-9-Proc: 53.42, 53.43, 53.62, 53.63, 54.21, 54.51, 59.03, 59.12 - ICD-9-Dx: V64.41 - Not robotic   Open:   - If none of the above |
| Partial Nephrectomy for Kidney Cancer | Partial Nephrectomy Procedure:   - CPT/HCPCS: 50543 - ICD-9-Proc: 55.4 (P)   AND  Kidney Cancer:   - ICD-9-Dx: 158.0, 189.0, 189.8, 189.9, 198.0, 236.91 | Radical Nephrectomy:   - ICD-9-Proc: 55.5X | Robotic:   - CPT/HCPCS: S2900 - ICD-9-Proc: 17.4X - Robotic billing record   Laparoscopic:   - CPT/HCPCS: 50543 - ICD-9-Proc: 54.21 - ICD-9-Dx: V64.41 - Not robotic   Open:   - If none of the above |

^1^ P: Primary procedure; O: Outpatient encounter
^2^ Robotic billing procedure identified using the following search patterns: '%ENDO%WRIST%','%ENDO%WRST%','%ENDWRST%','%ENDOWRST%','%ENDOWRIST%' ,'%ROBOT%','%INTUITIVE%','%VINCI%'
3Hand assist billing procedures identified using the following search patterns: '%HANDPORT%','%HAND%ASST%,'%GEL%PORT%,'%GELPORT%','%LAP%HAND%ASST%'
