# Supplementary material for: Impact of type of minimally invasive approach on open conversions across ten common procedures in different specialties
Source: Surg Endosc. 2022 Feb 9;36(8):6067–75. doi: 10.1007/s00464-022-09073-5 (PMC9283176; doi:10.1007/s00464-022-09073-5)
Supplement: Supplementary file 6 — Supplementary file6 (DOCX 101 KB) [file 464_2022_9073_MOESM6_ESM.docx]

Supplementary Table 2A. Characteristics of patients underwent hysterectomy for benign condition

|  | **Before Propensity Score Matching** | | | **After Propensity Score Matching^1^** | | |
| --- | --- | --- | --- | --- | --- | --- |
| **Characteristic** | **Lap N = 66367** | **RAS N = 61250** | **SD** | **Lap  N = 47673** | **RAS N = 47673** | **SD** |
| **Age (years), categorical** |  |  |  |  |  |  |
| 18-55 | 58303 (87.85) | 50834 (82.99) | -0.14 | 40907 (85.81) | 40511 (84.98) | -0.02 |
| 56-65 | 5324 (8.02) | 6536 (10.67) | 0.09 | 4399 (9.23) | 4658 (9.77) | 0.02 |
| 66+ | 2740 (4.13) | 3880 (6.33) | 0.1 | 2367 (4.97) | 2504 (5.25) | 0.01 |
| **Race** |  |  |  |  |  |  |
| White | 49047 (73.9) | 45304 (73.97) | 0.00 | 34889 (73.18) | 35055 (73.53) | 0.01 |
| Black | 8318 (12.53) | 7595 (12.4) | 0.00 | 6341 (13.3) | 6256 (13.12) | -0.01 |
| Other | 9002 (13.56) | 8351 (13.63) | 0.00 | 6443 (13.51) | 6362 (13.35) | 0 |
| **Primary Payor** |  |  |  |  |  |  |
| Commercial | 46344 (69.83) | 43817 (71.54) | 0.04 | 34457 (72.28) | 34083 (71.49) | -0.02 |
| Medicare | 5044 (7.6) | 6226 (10.16) | 0.09 | 4027 (8.45) | 4191 (8.79) | 0.01 |
| Medicaid | 9004 (13.57) | 6450 (10.53) | -0.09 | 5398 (11.32) | 5427 (11.38) | 0.00 |
| Other | 5975 (9) | 4757 (7.77) | -0.04 | 3791 (7.95) | 3972 (8.33) | 0.01 |
| **BMI >= 30** | 8658 (13.05) | 9391 (15.33) | 0.07 | 6772 (14.21) | 6795 (14.25) | 0.00 |
| **CCI>=1** | 12598 (18.98) | 12715 (20.76) | 0.04 | 9321 (19.55) | 9470 (19.86) | 0.01 |
| **Inpatient** | 16685 (25.14) | 13494 (22.03) | -0.07 | 10778 (22.61) | 11005 (23.08) | 0.01 |
| **Provider Region** |  |  |  |  |  |  |
| Midwest | 11664 (17.57) | 13439 (21.94) | 0.11 | 9260 (19.42) | 9192 (19.28) | 0.00 |
| Northeast | 4301 (6.48) | 4702 (7.68) | 0.05 | 3281 (6.88) | 3212 (6.74) | -0.01 |
| South | 33286 (50.15) | 28536 (46.59) | -0.07 | 23382 (49.05) | 23250 (48.77) | -0.01 |
| West | 17116 (25.79) | 14573 (23.79) | -0.05 | 11750 (24.65) | 12019 (25.21) | 0.01 |
| **Urban Hospital** | 58124 (87.58) | 57980 (94.66) | 0.25 | 44984 (94.36) | 44412 (93.16) | -0.05 |
| **Number of Hospital Beds** |  |  |  |  |  |  |
| 0-199 beds | 17028 (25.66) | 7682 (12.54) | -0.34 | 7813 (16.39) | 7623 (15.99) | -0.01 |
| 200-499 beds | 32303 (48.67) | 34871 (56.93) | 0.17 | 26439 (55.46) | 26477 (55.54) | 0.00 |
| 500+ beds | 17036 (25.67) | 18697 (30.53) | 0.11 | 13421 (28.15) | 13573 (28.47) | 0.01 |
| **Teaching hospital** | 21054 (31.72) | 21039 (34.35) | 0.06 | 15254 (32) | 15646 (32.82) | 0.02 |
| **Physician Volume** |  |  |  |  |  |  |
| Low | 19821 (29.87) | 10370 (16.93) | -0.31 | 11216 (23.53) | 10241 (21.48) | -0.05 |
| Moderate | 23761 (35.8) | 17755 (28.99) | -0.15 | 16498 (34.61) | 17019 (35.7) | 0.02 |
| High | 22785 (34.33) | 33125 (54.08) | 0.41 | 19959 (41.87) | 20413 (42.82) | 0.02 |
| **Physician Specialty** |  |  |  |  |  |  |
| Obstetrics/Gynecology | 59438 (89.56) | 51789 (84.55) | -0.15 | 42238 (88.6) | 42252 (88.63) | 0.00 |
| Gynecological Oncology | 2617 (3.94) | 5836 (9.53) | 0.22 | 2565 (5.38) | 2437 (5.11) | -0.01 |
| Other | 4312 (6.5) | 3625 (5.92) | -0.02 | 2870 (6.02) | 2984 (6.26) | 0.01 |
| **Procedure Year** |  |  |  |  |  |  |
| 2013 | 24313 (36.63) | 22907 (37.4) | 0.02 | 17700 (37.13) | 17136 (35.94) | -0.02 |
| 2014 | 25237 (38.03) | 22319 (36.44) | -0.03 | 17597 (36.91) | 18028 (37.82) | 0.02 |
| 2015 | 16817 (25.34) | 16024 (26.16) | 0.02 | 12376 (25.96) | 12509 (26.24) | 0.01 |

Abbreviation: BMI, body mass index; CCI, Charlson comorbidity index; Lap, Laparoscopic; RAS, robotic-assisted Surgery; SD, standard difference
1. 1:1 propensity score matching using the 5-to-1-digit greedy matching algorithm.

Supplementary Table 2B. Characteristics of patients underwent hysterectomy for endometrial cancer

|  | **Before Propensity Score Matching** | | | **After Propensity Score Matching**^1^ | | |
| --- | --- | --- | --- | --- | --- | --- |
| **Characteristic** | **Lap N = 1578** | **RAS N = 8356** | **SD** | **Lap  N = 987** | **RAS N = 2961** | **SD** |
| **Age (years), categorical** |  |  |  |  |  |  |
| 18-55 | 372 (23.57) | 1921 (22.99) | -0.01 | 219 (22.19) | 679 (22.93) | 0.02 |
| 56-65 | 561 (35.55) | 3127 (37.42) | 0.04 | 337 (34.14) | 1106 (37.35) | 0.07 |
| 66+ | 645 (40.87) | 3308 (39.59) | -0.03 | 431 (43.67) | 1176 (39.72) | -0.08 |
| **Race** |  |  |  |  |  |  |
| White | 1229 (77.88) | 6736 (80.61) | 0.07 | 776 (78.62) | 2294 (77.47) | -0.03 |
| Black | 121 (7.67) | 488 (5.84) | -0.07 | 72 (7.29) | 199 (6.72) | -0.02 |
| Other | 228 (14.45) | 1132 (13.55) | -0.03 | 139 (14.08) | 468 (15.81) | 0.05 |
| **Primary Payor** |  |  |  |  |  |  |
| Commercial | 642 (40.68) | 3723 (44.55) | 0.08 | 412 (41.74) | 1246 (42.08) | 0.01 |
| Medicare | 732 (46.39) | 3627 (43.41) | -0.06 | 474 (48.02) | 1347 (45.49) | -0.05 |
| Medicaid | 102 (6.46) | 489 (5.85) | -0.03 | 54 (5.47) | 188 (6.35) | 0.04 |
| Other | 102 (6.46) | 517 (6.19) | -0.01 | 47 (4.76) | 180 (6.08) | 0.06 |
| **BMI >= 30** | 477 (30.23) | 3258 (38.99) | 0.18 | 323 (32.73) | 892 (30.12) | -0.06 |
| **CCI>=1** | 683 (43.28) | 3637 (43.53) | 0.00 | 401 (40.63) | 1196 (40.39) | 0.00 |
| **Inpatient** | 878 (55.64) | 3890 (46.55) | -0.18 | 473 (47.92) | 1490 (50.32) | 0.05 |
| **Provider Region** |  |  |  |  |  |  |
| Midwest | 287 (18.19) | 1486 (17.78) | -0.01 | 199 (20.16) | 582 (19.66) | -0.01 |
| Northeast | 177 (11.22) | 1297 (15.52) | 0.13 | 123 (12.46) | 321 (10.84) | -0.05 |
| South | 746 (47.28) | 3299 (39.48) | -0.16 | 404 (40.93) | 1268 (42.82) | 0.04 |
| West | 368 (23.32) | 2274 (27.21) | 0.09 | 261 (26.44) | 790 (26.68) | 0.01 |
| **Urban Hospital** | 73 (4.63) | 65 (0.78) | -0.24 | 9 (0.91) | 24 (0.81) | -0.01 |
| **Number of Hospital Beds** |  |  |  |  |  |  |
| 0-199 beds | 297 (18.82) | 376 (4.5) | -0.46 | 65 (6.59) | 192 (6.48) | 0.00 |
| 200-499 beds | 556 (35.23) | 3800 (45.48) | 0.21 | 406 (41.13) | 1196 (40.39) | -0.02 |
| 500+ beds | 725 (45.94) | 4180 (50.02) | 0.08 | 516 (52.28) | 1573 (53.12) | 0.02 |
| **Teaching Hospital** | 858 (54.37) | 4689 (56.12) | 0.04 | 595 (60.28) | 1814 (61.26) | 0.02 |
| **Physician Volume** |  |  |  |  |  |  |
| Low | 722 (45.75) | 1577 (18.87) | -0.6 | 371 (37.59) | 963 (32.52) | -0.11 |
| Moderate | 509 (32.26) | 2924 (34.99) | 0.06 | 383 (38.8) | 1219 (41.17) | 0.05 |
| High | 347 (21.99) | 3855 (46.13) | 0.53 | 233 (23.61) | 779 (26.31) | 0.06 |
| **Physician Specialty** |  |  |  |  |  |  |
| Obstetrics/Gynecology | 400 (25.35) | 2773 (33.19) | 0.17 | 310 (31.41) | 856 (28.91) | -0.05 |
| Gyn Oncology | 1005 (63.69) | 5069 (60.66) | -0.06 | 604 (61.2) | 1868 (63.09) | 0.04 |
| Other | 173 (10.96) | 514 (6.15) | -0.17 | 73 (7.4) | 237 (8) | 0.02 |
| **Procedure year** |  |  |  |  |  |  |
| 2013 | 460 (29.15) | 2862 (34.25) | 0.11 | 313 (31.71) | 861 (29.08) | -0.06 |
| 2014 | 631 (39.99) | 2970 (35.54) | -0.09 | 386 (39.11) | 1147 (38.74) | -0.01 |
| 2015 | 487 (30.86) | 2524 (30.21) | -0.01 | 288 (29.18) | 953 (32.19) | 0.07 |

Abbreviation: BMI, body mass index; CCI, Charlson comorbidity index; Lap, Laparoscopic; RAS, robotic-assisted Surgery; SD, standard difference
1. 1:3 propensity score matching using the 8-to-1-digit matching algorithm.

Supplementary Table 2C. Characteristics of patients underwent right colectomy for benign conditions

|  | **Before Propensity Score Matching** | | | **After Propensity Score Matching**^1^ | | |
| --- | --- | --- | --- | --- | --- | --- |
| **Characteristic** | **Lap N = 11431** | **RAS N = 1187** | **SD** | **Lap N = 1187** | **RAS N = 1187** | **SD** |
| **Age (years), categorical** |  |  |  |  |  |  |
| 18-55 | 2962 (25.91) | 267 (22.49) | -0.08 | 232 (19.55) | 267 (22.49) | 0.07 |
| 56-65 | 3056 (26.73) | 333 (28.05) | 0.03 | 340 (28.64) | 333 (28.05) | -0.01 |
| 66+ | 5413 (47.35) | 587 (49.45) | 0.04 | 615 (51.81) | 587 (49.45) | -0.05 |
| **Gender** |  |  |  |  |  |  |
| Male | 5433 (47.53) | 593 (49.96) | 0.05 | 571 (48.1) | 593 (49.96) | 0.04 |
| Female | 5998 (52.47) | 594 (50.04) | -0.05 | 616 (51.9) | 594 (50.04) | -0.04 |
| **Race** |  |  |  |  |  |  |
| White | 8982 (78.58) | 936 (78.85) | 0.01 | 937 (78.94) | 936 (78.85) | 0.00 |
| Black | 1267 (11.08) | 110 (9.27) | -0.06 | 129 (10.87) | 110 (9.27) | -0.05 |
| Other | 1182 (10.34) | 141 (11.88) | 0.05 | 121 (10.19) | 141 (11.88) | 0.05 |
| **Primary Payor** |  |  |  |  |  |  |
| Commercial | 4639 (40.58) | 487 (41.03) | 0.01 | 464 (39.09) | 487 (41.03) | 0.04 |
| Medicare | 5784 (50.6) | 616 (51.9) | 0.03 | 656 (55.27) | 616 (51.9) | -0.07 |
| Medicaid | 537 (4.7) | 47 (3.96) | -0.04 | 33 (2.78) | 47 (3.96) | 0.07 |
| Other | 471 (4.12) | 37 (3.12) | -0.05 | 34 (2.86) | 37 (3.12) | 0.01 |
| **BMI >= 30** | 1754 (15.34) | 177 (14.91) | -0.01 | 171 (14.41) | 177 (14.91) | 0.01 |
| **CCI>=1** | 1754 (15.34) | 177 (14.91) | -0.01 | 171 (14.41) | 177 (14.91) | 0.01 |
| **Inpatient**^2^ | 11431 (100) | 1187 (100) | 0.00 | 1187 (100) | 1187 (100) | 0.00 |
| **Provider Region** |  |  |  |  |  |  |
| Midwest | 2409 (21.07) | 227 (19.12) | -0.05 | 243 (20.47) | 227 (19.12) | -0.03 |
| Northeast | 1646 (14.4) | 207 (17.44) | 0.08 | 187 (15.75) | 207 (17.44) | 0.05 |
| South | 5305 (46.41) | 549 (46.25) | 0.00 | 595 (50.13) | 549 (46.25) | -0.08 |
| West | 2071 (18.12) | 204 (17.19) | -0.02 | 162 (13.65) | 204 (17.19) | 0.1 |
| **Urban Hospital** | 10338 (90.44) | 1119 (94.27) | 0.14 | 1125 (94.78) | 1119 (94.27) | -0.02 |
| **Number of Hospital Beds** |  |  |  |  |  |  |
| 0-199 beds | 1825 (15.97) | 137 (11.54) | -0.13 | 139 (11.71) | 137 (11.54) | -0.01 |
| 200-499 beds | 5472 (47.87) | 643 (54.17) | 0.13 | 637 (53.66) | 643 (54.17) | 0.01 |
| 500+ beds | 4134 (36.16) | 407 (34.29) | -0.04 | 411 (34.63) | 407 (34.29) | -0.01 |
| **Teaching Hospital** | 4876 (42.66) | 436 (36.73) | -0.12 | 410 (34.54) | 436 (36.73) | 0.05 |
| **Physician Volume** |  |  |  |  |  |  |
| Low | 3797 (33.22) | 610 (51.39) | 0.37 | 594 (50.04) | 610 (51.39) | 0.03 |
| Moderate | 3343 (29.25) | 307 (25.86) | -0.08 | 326 (27.46) | 307 (25.86) | -0.04 |
| High | 4291 (37.54) | 270 (22.75) | -0.33 | 267 (22.49) | 270 (22.75) | 0.01 |
| **Physician Specialty** |  |  |  |  |  |  |
| Colon/Rectal Surgery | 3157 (27.62) | 400 (33.7) | 0.13 | 403 (33.95) | 400 (33.7) | -0.01 |
| Others | 8274 (72.38) | 787 (66.3) | -0.13 | 784 (66.05) | 787 (66.3) | 0.01 |
| **Procedure year** |  |  |  |  |  |  |
| 2013 | 4092 (35.8) | 316 (26.62) | -0.2 | 330 (27.8) | 316 (26.62) | -0.03 |
| 2014 | 4281 (37.45) | 446 (37.57) | 0.00 | 429 (36.14) | 446 (37.57) | 0.03 |
| 2015 | 3058 (26.75) | 425 (35.8) | 0.20 | 428 (36.06) | 425 (35.8) | -0.01 |

Abbreviation: BMI, body mass index; CCI, Charlson comorbidity index; Lap, Laparoscopic; RAS, robotic-assisted Surgery; SD, standard difference
1. 1:1 propensity score matching using the 5-to-1-digit greedy matching algorithm.
2. The characteristic is not used in propensity score matching

Supplementary Table 2D. Characteristics of patients underwent right colectomy for malignant conditions

|  | **Before Propensity Score Matching** | | | **After Propensity Score Matching**^1^ | | |
| --- | --- | --- | --- | --- | --- | --- |
| **Characteristic** | **Lap N = 7693** | **RAS N = 829** | **SD** | **Lap N = 829** | **RAS N = 829** | **SD** |
| **Age (years), categorical** |  |  |  |  |  |  |
| 18-55 | 936 (12.17) | 121 (14.6) | 0.07 | 113 (13.63) | 121 (14.6) | 0.03 |
| 56-65 | 1578 (20.51) | 178 (21.47) | 0.02 | 150 (18.09) | 178 (21.47) | 0.08 |
| 66+ | 5179 (67.32) | 530 (63.93) | -0.07 | 566 (68.28) | 530 (63.93) | -0.09 |
| **Gender/Male** | 3494 (45.42) | 407 (49.1) | 0.07 | 396 (47.77) | 407 (49.1) | 0.03 |
| **Race** |  |  |  |  |  |  |
| White | 6036 (78.46) | 639 (77.08) | -0.03 | 643 (77.56) | 639 (77.08) | -0.01 |
| Black | 714 (9.28) | 78 (9.41) | 0 | 76 (9.17) | 78 (9.41) | 0.01 |
| Other | 943 (12.26) | 112 (13.51) | 0.04 | 110 (13.27) | 112 (13.51) | 0.01 |
| **Primary Payor** |  |  |  |  |  |  |
| Commercial | 2075 (26.97) | 268 (32.33) | 0.12 | 257 (31) | 268 (32.33) | 0.03 |
| Medicare | 5143 (66.85) | 510 (61.52) | -0.11 | 537 (64.78) | 510 (61.52) | -0.07 |
| Medicaid | 242 (3.15) | 24 (2.9) | -0.01 | 17 (2.05) | 24 (2.9) | 0.05 |
| Other | 233 (3.03) | 27 (3.26) | 0.01 | 18 (2.17) | 27 (3.26) | 0.07 |
| **BMI >= 30** | 1116 (14.51) | 143 (17.25) | 0.08 | 131 (15.8) | 143 (17.25) | 0.04 |
| **CCI>=3** | 4577 (59.5) | 456 (55.01) | -0.09 | 479 (57.78) | 456 (55.01) | -0.06 |
| **Inpatient**^2^ | 7693 (100) | 829 (100) | 0.00 | 829 (100) | 829 (100) | 0.00 |
| **Provider Region** |  |  |  |  |  |  |
| Midwest | 1608 (20.9) | 136 (16.41) | -0.12 | 147 (17.73) | 136 (16.41) | -0.04 |
| Northeast | 1212 (15.75) | 160 (19.3) | 0.09 | 140 (16.89) | 160 (19.3) | 0.06 |
| South | 3398 (44.17) | 364 (43.91) | -0.01 | 366 (44.15) | 364 (43.91) | 0.00 |
| West | 1475 (19.17) | 169 (20.39) | 0.03 | 176 (21.23) | 169 (20.39) | -0.02 |
| **Urban Hospital** | 6891 (89.57) | 771 (93) | 0.12 | 770 (92.88) | 771 (93.00) | 0.00 |
| **Number of Hospital Beds** |  |  |  |  |  |  |
| 0-199 beds | 1256 (16.33) | 75 (9.05) | -0.22 | 65 (7.84) | 75 (9.05) | 0.04 |
| 200-499 beds | 3724 (48.41) | 474 (57.18) | 0.18 | 477 (57.54) | 474 (57.18) | -0.01 |
| 500+ beds | 2713 (35.27) | 280 (33.78) | -0.03 | 287 (34.62) | 280 (33.78) | -0.02 |
| **Teaching hospital** | 3314 (43.08) | 309 (37.27) | -0.12 | 301 (36.31) | 309 (37.27) | 0.02 |
| **Physician Volume** |  |  |  |  |  |  |
| Low | 1858 (24.15) | 316 (38.12) | 0.31 | 336 (40.53) | 316 (38.12) | -0.05 |
| Moderate | 3229 (41.97) | 320 (38.6) | -0.07 | 311 (37.52) | 320 (38.6) | 0.02 |
| High | 2606 (33.87) | 193 (23.28) | -0.24 | 182 (21.95) | 193 (23.28) | 0.03 |
| **Physician Specialty** |  |  |  |  |  |  |
| Colon/Rectal Surgery | 1993 (25.91) | 284 (34.26) | 0.18 | 275 (33.17) | 284 (34.26) | 0.02 |
| General Surgery and other | 5700 (74.09) | 545 (65.74) | -0.18 | 554 (66.83) | 545 (65.74) | -0.02 |
| **Procedure year** |  |  |  |  |  |  |
| 2013 | 2856 (37.12) | 216 (26.06) | -0.24 | 216 (26.06) | 216 (26.06) | 0 |
| 2014 | 2783 (36.18) | 323 (38.96) | 0.06 | 344 (41.5) | 323 (38.96) | -0.05 |
| 2015 | 2054 (26.7) | 290 (34.98) | 0.18 | 269 (32.45) | 290 (34.98) | 0.05 |

Abbreviation: BMI, body mass index; CCI, Charlson comorbidity index; Lap, Laparoscopic; RAS, robotic-assisted Surgery; SD, standard difference
1. 1:1 propensity score matching using the 5-to-1-digit greedy matching algorithm.
2. The characteristic is not used in propensity score matching

Supplementary Table 2E. Characteristics of patients underwent sigmoidectomy for diverticular disease

|  | **Before Propensity Score Matching** | | | **After Propensity Score Matching**^1^ | | |
| --- | --- | --- | --- | --- | --- | --- |
| **Characteristic** | **Lap  N = 11832** | **RAS N = 1963** | **SD** | **Lap  N = 1963** | **RAS N = 1963** | **SD** |
| **Age (years), categorical** |  |  |  |  |  |  |
| 18-55 | 5105 (43.15) | 881 (44.88) | 0.03 | 860 (43.81) | 881 (44.88) | 0.02 |
| 56-65 | 3540 (29.92) | 583 (29.7) | 0 | 571 (29.09) | 583 (29.7) | 0.01 |
| 66+ | 3187 (26.94) | 499 (25.42) | -0.03 | 532 (27.1) | 499 (25.42) | -0.04 |
| **Gender/Male** | 5331 (45.06) | 874 (44.52) | -0.01 | 878 (44.73) | 874 (44.52) | 0.00 |
| **Race** |  |  |  |  |  |  |
| White | 10034 (84.8) | 1653 (84.21) | -0.02 | 1700 (86.6) | 1653 (84.21) | -0.07 |
| Black | 565 (4.78) | 93 (4.74) | 0 | 69 (3.52) | 93 (4.74) | 0.06 |
| Other | 1233 (10.42) | 217 (11.05) | 0.02 | 194 (9.88) | 217 (11.05) | 0.04 |
| **Primary Payor** |  |  |  |  |  |  |
| Commercial | 6842 (57.83) | 1183 (60.26) | 0.05 | 1216 (61.95) | 1183 (60.26) | -0.03 |
| Medicare | 3645 (30.81) | 566 (28.83) | -0.04 | 573 (29.19) | 566 (28.83) | -0.01 |
| Medicaid | 630 (5.32) | 107 (5.45) | 0.01 | 96 (4.89) | 107 (5.45) | 0.03 |
| Other | 715 (6.04) | 107 (5.45) | -0.03 | 78 (3.97) | 107 (5.45) | 0.07 |
| **BMI >= 30** | 2066 (17.46) | 331 (16.86) | -0.02 | 300 (15.28) | 331 (16.86) | 0.04 |
| **CCI>=1** | 3799 (32.11) | 590 (30.06) | -0.04 | 579 (29.5) | 590 (30.06) | 0.01 |
| **Inpatient**^2^ | 11832 (100) | 1963 (100) | 0.00 | 1963 (100) | 1963 (100) | 0.00 |
| **Provider Region** |  |  |  |  |  |  |
| Midwest | 2807 (23.72) | 379 (19.31) | -0.11 | 384 (19.56) | 379 (19.31) | -0.01 |
| Northeast | 2019 (17.06) | 401 (20.43) | 0.09 | 442 (22.52) | 401 (20.43) | -0.05 |
| South | 5001 (42.27) | 821 (41.82) | -0.01 | 792 (40.35) | 821 (41.82) | 0.03 |
| West | 2005 (16.95) | 362 (18.44) | 0.04 | 345 (17.58) | 362 (18.44) | 0.02 |
| **Urban Hospital** | 10774 (91.06) | 1874 (95.47) | 0.18 | 1876 (95.57) | 1874 (95.47) | 0.00 |
| **Number of Hospital Beds** |  |  |  |  |  |  |
| 0-199 beds | 2411 (20.38) | 268 (13.65) | -0.18 | 267 (13.6) | 268 (13.65) | 0.00 |
| 200-499 beds | 5630 (47.58) | 991 (50.48) | 0.06 | 990 (50.43) | 991 (50.48) | 0.00 |
| 500+ beds | 3791 (32.04) | 704 (35.86) | 0.08 | 706 (35.97) | 704 (35.86) | 0.00 |
| **Teaching hospital** | 4701 (39.73) | 801 (40.8) | 0.02 | 825 (42.03) | 801 (40.8) | -0.02 |
| **Physician Volume** |  |  |  |  |  |  |
| Low | 3846 (32.51) | 786 (40.04) | 0.16 | 761 (38.77) | 786 (40.04) | 0.03 |
| Moderate | 3560 (30.09) | 520 (26.49) | -0.08 | 556 (28.32) | 520 (26.49) | -0.04 |
| High | 4426 (37.41) | 657 (33.47) | -0.08 | 646 (32.91) | 657 (33.47) | 0.01 |
| **Physician Specialty** |  |  |  |  |  |  |
| Colon/Rectal Surgery | 2628 (22.21) | 689 (35.1) | 0.29 | 665 (33.88) | 689 (35.1) | 0.03 |
| General Surgery &Other | 9204 (77.79) | 1274 (64.9) | -0.29 | 1298 (66.12) | 1274 (64.9) | -0.03 |
| **Procedure year** |  |  |  |  |  |  |
| 2013 | 4439 (37.52) | 489 (24.91) | -0.27 | 482 (24.55) | 489 (24.91) | 0.01 |
| 2014 | 4323 (36.54) | 746 (38) | 0.03 | 721 (36.73) | 746 (38) | 0.03 |
| 2015 | 3070 (25.95) | 728 (37.09) | 0.24 | 760 (38.72) | 728 (37.09) | -0.03 |

Abbreviation: BMI, body mass index; CCI, Charlson comorbidity index; Lap, Laparoscopic; RAS, robotic-assisted Surgery; SD, standard difference
1. 1:1 propensity score matching using the 5-to-1-digit greedy matching algorithm.
2. The characteristic is not used in propensity score matching

Supplementary Table 2F. Characteristics of patients underwent low anterior resection for rectal cancer

|  | **Before Propensity Score Matching** | | | **After Propensity Score Matching**^1^ | | |
| --- | --- | --- | --- | --- | --- | --- |
| **Characteristic** | **Lap  N = 1573** | **RAS N = 1290** | **SD** | **Lap  N = 940** | **RAS N = 940** | **SD** |
| **Age (years), categorical** | |  |  |  |  |  |
| 18-55 | 501 (31.85) | 426 (33.02) | 0.03 | 311 (33.09) | 312 (33.19) | 0.00 |
| 56-65 | 421 (26.76) | 405 (31.4) | 0.10 | 268 (28.51) | 266 (28.3) | 0.00 |
| 66+ | 651 (41.39) | 459 (35.58) | -0.12 | 361 (38.4) | 362 (38.51) | 0.00 |
| **Gender** |  |  |  |  |  |  |
| Male | 902 (57.34) | 749 (58.06) | 0.01 | 530 (56.38) | 546 (58.09) | 0.03 |
| Female | 671 (42.66) | 541 (41.94) | -0.01 | 410 (43.62) | 394 (41.91) | -0.03 |
| **Race** |  |  |  |  |  |  |
| White | 1233 (78.39) | 1034 (80.16) | 0.04 | 747 (79.47) | 746 (79.36) | 0.00 |
| Black | 113 (7.18) | 79 (6.12) | -0.04 | 61 (6.49) | 65 (6.91) | 0.02 |
| Other | 227 (14.43) | 177 (13.72) | -0.02 | 132 (14.04) | 129 (13.72) | -0.01 |
| **Primary Payor** |  |  |  |  |  |  |
| Commercial | 717 (45.58) | 638 (49.46) | 0.08 | 462 (49.15) | 464 (49.36) | 0.00 |
| Medicare | 675 (42.91) | 486 (37.67) | -0.11 | 369 (39.26) | 373 (39.68) | 0.01 |
| Medicaid | 98 (6.23) | 106 (8.22) | 0.08 | 64 (6.81) | 60 (6.38) | -0.02 |
| Other | 83 (5.28) | 60 (4.65) | -0.03 | 45 (4.79) | 43 (4.57) | -0.01 |
| **BMI >= 30** | 268 (17.04) | 174 (13.49) | -0.10 | 138 (14.68) | 137 (14.57) | 0.00 |
| **CCI>=3** | 836 (53.15) | 643 (49.84) | -0.07 | 486 (51.7) | 487 (51.81) | 0.00 |
| **Inpatient**^2^ | 1573 (100) | 1290 (100) | 0.00 | 940 (100) | 940 (100) | 0.00 |
| **Provider Region** |  |  |  |  |  |  |
| Midwest | 265 (16.85) | 269 (20.85) | 0.10 | 194 (20.64) | 196 (20.85) | 0.01 |
| Northeast | 270 (17.16) | 277 (21.47) | 0.11 | 166 (17.66) | 175 (18.62) | 0.02 |
| South | 679 (43.17) | 538 (41.71) | -0.03 | 397 (42.23) | 393 (41.81) | -0.01 |
| West | 359 (22.82) | 206 (15.97) | -0.17 | 183 (19.47) | 176 (18.72) | -0.02 |
| **Urban Hospital** | 1405 (89.32) | 1249 (96.82) | 0.30 | 895 (95.21) | 899 (95.64) | 0.02 |
| **Number of Hospital Beds** | |  |  |  |  |  |
| 0-199 beds | 196 (12.46) | 100 (7.75) | -0.16 | 96 (10.21) | 87 (9.26) | -0.03 |
| 200-499 beds | 728 (46.28) | 619 (47.98) | 0.03 | 467 (49.68) | 476 (50.64) | 0.02 |
| 500+ beds | 649 (41.26) | 571 (44.26) | 0.06 | 377 (40.11) | 377 (40.11) | 0.00 |
| **Teaching hospital** | 703 (44.69) | 702 (54.42) | 0.20 | 467 (49.68) | 466 (49.57) | 0.00 |
| **Physician Volume** |  |  |  |  |  |  |
| Low | 780 (49.59) | 364 (28.22) | -0.45 | 371 (39.47) | 362 (38.51) | -0.02 |
| Moderate | 533 (33.88) | 390 (30.23) | -0.08 | 330 (35.11) | 357 (37.98) | 0.06 |
| High | 260 (16.53) | 536 (41.55) | 0.57 | 239 (25.43) | 221 (23.51) | -0.04 |
| **Physician Specialty** |  |  |  |  |  |  |
| Colon/Rectal Surgery | 598 (38.02) | 688 (53.33) | 0.31 | 440 (46.81) | 463 (49.26) | 0.05 |
| General Surgery and Other | 975 (61.98) | 602 (46.67) | -0.31 | 500 (53.19) | 477 (50.74) | -0.05 |
| **Procedure year** |  |  |  |  |  |  |
| 2013 | 542 (34.46) | 328 (25.43) | -0.20 | 281 (29.89) | 279 (29.68) | 0.00 |
| 2014 | 614 (39.03) | 501 (38.84) | 0.00 | 358 (38.09) | 359 (38.19) | 0.00 |
| 2015 | 417 (26.51) | 461 (35.74) | 0.20 | 301 (32.02) | 302 (32.13) | 0.00 |

Abbreviation: BMI, body mass index; CCI, Charlson comorbidity index; Lap, Laparoscopic; RAS, robotic-assisted Surgery; SD, standard difference
1. 1:1 propensity score matching using the 5-to-1-digit greedy matching algorithm.
2. The characteristic is not used in propensity score matching

Supplementary Table 2F. Characteristics of patients underwent inguinal hernia repairs

|  | **Before Propensity Score Matching** | | | **After Propensity Score Matching**^1^ | | |
| --- | --- | --- | --- | --- | --- | --- |
| **Characteristic** | **Lap N = 46821** | **RAS N = 3692** | **SD** | **Lap N = 3692** | **RAS N = 3692** | **SD** |
| **Age (years), categorical** |  |  |  |  |  |  |
| 18-55 | 21520 (45.96) | 1686 (45.67) | -0.01 | 1796 (48.65) | 1686 (45.67) | -0.06 |
| 56-65 | 11731 (25.05) | 920 (24.92) | 0.00 | 771 (20.88) | 920 (24.92) | 0.10 |
| 66+ | 13570 (28.98) | 1086 (29.41) | 0.01 | 1125 (30.47) | 1086 (29.41) | -0.02 |
| **Gender** |  |  |  |  |  |  |
| Male | 43568 (93.05) | 3399 (92.06) | -0.04 | 3387 (91.74) | 3399 (92.06) | 0.01 |
| Female | 3253 (6.95) | 293 (7.94) | 0.04 | 305 (8.26) | 293 (7.94) | -0.01 |
| **Race** |  |  |  |  |  |  |
| White | 38847 (82.97) | 3042 (82.39) | -0.02 | 3003 (81.34) | 3042 (82.39) | 0.03 |
| Black | 2367 (5.06) | 256 (6.93) | 0.08 | 271 (7.34) | 256 (6.93) | -0.02 |
| Other | 5607 (11.98) | 394 (10.67) | -0.04 | 418 (11.32) | 394 (10.67) | -0.02 |
| **Primary Payor** |  |  |  |  |  |  |
| Commercial | 24704 (52.76) | 1954 (52.93) | 0.00 | 1934 (52.38) | 1954 (52.93) | 0.01 |
| Medicare | 13893 (29.67) | 1092 (29.58) | 0.00 | 1133 (30.69) | 1092 (29.58) | -0.02 |
| Medicaid | 3039 (6.49) | 316 (8.56) | 0.08 | 325 (8.8) | 316 (8.56) | -0.01 |
| Other | 5185 (11.07) | 330 (8.94) | -0.07 | 300 (8.13) | 330 (8.94) | 0.03 |
| **BMI≥30** | 1830 (3.91) | 165 (4.47) | 0.03 | 134 (3.63) | 165 (4.47) | 0.04 |
| **CCI≥1** | 9508 (20.31) | 809 (21.91) | 0.04 | 832 (22.54) | 809 (21.91) | -0.01 |
| **Inpatient^2^** | 831 (1.77) | 99 (2.68) | 0.06 | 60 (1.63) | 99 (2.68) | 0.07 |
| **Provider Region** |  |  |  |  |  |  |
| Midwest | 11403 (24.35) | 599 (16.22) | -0.20 | 625 (16.93) | 599 (16.22) | -0.02 |
| Northeast | 4454 (9.51) | 230 (6.23) | -0.12 | 236 (6.39) | 230 (6.23) | -0.01 |
| South | 18433 (39.37) | 1631 (44.18) | 0.10 | 1582 (42.85) | 1631 (44.18) | 0.03 |
| West | 12531 (26.76) | 1232 (33.37) | 0.14 | 1249 (33.83) | 1232 (33.37) | -0.01 |
| **Urban Hospital** | 41416 (88.46) | 3462 (93.77) | 0.19 | 3497 (94.72) | 3462 (93.77) | -0.04 |
| **Number of Hospital Beds** |  |  |  |  |  |  |
| 0-199 beds | 11507 (24.58) | 761 (20.61) | -0.09 | 797 (21.59) | 761 (20.61) | -0.02 |
| 200-499 beds | 24200 (51.69) | 2063 (55.88) | 0.08 | 2094 (56.72) | 2063 (55.88) | -0.02 |
| 500+ beds | 11114 (23.74) | 868 (23.51) | -0.01 | 801 (21.7) | 868 (23.51) | 0.04 |
| **Teaching hospital** | 15631 (33.38) | 995 (26.95) | -0.14 | 967 (26.19) | 995 (26.95) | 0.02 |
| **Physician Volume** |  |  |  |  |  |  |
| Low | 15490 (33.08) | 2248 (60.89) | 0.58 | 2211 (59.89) | 2248 (60.89) | 0.02 |
| Moderate | 14637 (31.26) | 926 (25.08) | -0.14 | 936 (25.35) | 926 (25.08) | -0.01 |
| High | 16694 (35.65) | 518 (14.03) | -0.52 | 545 (14.76) | 518 (14.03) | -0.02 |
| **Procedure year** |  |  |  |  |  |  |
| 2013 | 16532 (35.31) | 281 (7.61) | -0.72 | 286 (7.75) | 281 (7.61) | -0.01 |
| 2014 | 17588 (37.56) | 1193 (32.31) | -0.11 | 1141 (30.9) | 1193 (32.31) | 0.03 |
| 2015 | 12701 (27.13) | 2218 (60.08) | 0.70 | 2265 (61.35) | 2218 (60.08) | -0.03 |

Abbreviation: BMI, body mass index; CCI, Charlson comorbidity index; Lap, Laparoscopic; RAS, robotic-assisted Surgery; SD, standard difference
1. 1:1 propensity score matching using the 5-to-1-digit greedy matching algorithm.
2. The characteristic is not used in propensity score matching

Supplementary Table 2G. Characteristics of patients underwent ventral hernia repairs

|  | **Before Propensity Score Matching** | | | **After Propensity Score Matching**^1^ | | |
| --- | --- | --- | --- | --- | --- | --- |
| **Characteristic** | **Lap  N = 36873** | **RAS N = 2703** | **SD** | **Lap**  **N = 2703** | **RAS N = 2703** | **SD** |
| **Age (years), categorical** |  |  |  |  |  |  |
| 18-55 | 18752 (50.86) | 1329 (49.17) | -0.03 | 1285 (47.54) | 1329 (49.17) | 0.03 |
| 56-65 | 8971 (24.33) | 702 (25.97) | 0.04 | 720 (26.64) | 702 (25.97) | -0.02 |
| 66+ | 9150 (24.81) | 672 (24.86) | 0.00 | 698 (25.82) | 672 (24.86) | -0.02 |
| **Gender** |  |  |  |  |  |  |
| Male | 17105 (46.39) | 1179 (43.62) | -0.06 | 1189 (43.99) | 1179 (43.62) | -0.01 |
| Female | 19768 (53.61) | 1524 (56.38) | 0.06 | 1514 (56.01) | 1524 (56.38) | 0.01 |
| **Race** |  |  |  |  |  |  |
| White | 29456 (79.89) | 2094 (77.47) | -0.06 | 2128 (78.73) | 2094 (77.47) | -0.03 |
| Black | 3658 (9.92) | 283 (10.47) | 0.02 | 307 (11.36) | 283 (10.47) | -0.03 |
| Other | 3759 (10.19) | 326 (12.06) | 0.06 | 268 (9.91) | 326 (12.06) | 0.07 |
| **Primary Payor** |  |  |  |  |  |  |
| Commercial | 17467 (47.37) | 1308 (48.39) | 0.02 | 1301 (48.13) | 1308 (48.39) | 0.01 |
| Medicare | 11867 (32.18) | 855 (31.63) | -0.01 | 847 (31.34) | 855 (31.63) | 0.01 |
| Medicaid | 4562 (12.37) | 343 (12.69) | 0.01 | 358 (13.24) | 343 (12.69) | -0.02 |
| Other | 2977 (8.07) | 197 (7.29) | -0.03 | 197 (7.29) | 197 (7.29) | 0.00 |
| **BMI≥30** | 9264 (25.12) | 634 (23.46) | -0.04 | 661 (24.45) | 634 (23.46) | -0.02 |
| **CCI≥1** | 14042 (38.08) | 1028 (38.03) | 0.00 | 1013 (37.48) | 1028 (38.03) | 0.01 |
| **Inpatient** | 8702 (23.6) | 760 (28.12) | 0.10 | 757 (28.01) | 760 (28.12) | 0.00 |
| **Provider Region** |  |  |  |  |  |  |
| Midwest | 8087 (21.93) | 437 (16.17) | -0.15 | 456 (16.87) | 437 (16.17) | -0.02 |
| Northeast | 3427 (9.29) | 245 (9.06) | -0.01 | 235 (8.69) | 245 (9.06) | 0.01 |
| South | 18469 (50.09) | 1256 (46.47) | -0.07 | 1263 (46.73) | 1256 (46.47) | -0.01 |
| West | 6890 (18.69) | 765 (28.3) | 0.23 | 749 (27.71) | 765 (28.3) | 0.01 |
| **Urban Hospital** | 32222 (87.39) | 2561 (94.75) | 0.26 | 2564 (94.86) | 2561 (94.75) | 0.00 |
| **Number of Hospital Beds** |  |  |  |  |  |  |
| 0-199 beds | 8664 (23.5) | 340 (12.58) | -0.29 | 357 (13.21) | 340 (12.58) | -0.02 |
| 200-499 beds | 18674 (50.64) | 1328 (49.13) | -0.03 | 1335 (49.39) | 1328 (49.13) | -0.01 |
| 500+ beds | 9535 (25.86) | 1035 (38.29) | 0.27 | 1011 (37.4) | 1035 (38.29) | 0.02 |
| **Teaching hospital** | 12639 (34.28) | 822 (30.41) | -0.08 | 853 (31.56) | 822 (30.41) | -0.02 |
| **Physician Volume** |  |  |  |  |  |  |
| Low | 16503 (44.76) | 1885 (69.74) | 0.52 | 1978 (73.18) | 1885 (69.74) | -0.08 |
| Moderate | 11838 (32.1) | 566 (20.94) | -0.25 | 491 (18.17) | 566 (20.94) | 0.07 |
| High | 8532 (23.14) | 252 (9.32) | -0.38 | 234 (8.66) | 252 (9.32) | 0.02 |
| **Procedure year** |  |  |  |  |  |  |
| 2013 | 13144 (35.65) | 345 (12.76) | -0.55 | 351 (12.99) | 345 (12.76) | -0.01 |
| 2014 | 13952 (37.84) | 978 (36.18) | -0.03 | 1005 (37.18) | 978 (36.18) | -0.02 |
| 2015 | 9777 (26.52) | 1380 (51.05) | 0.52 | 1347 (49.83) | 1380 (51.05) | 0.02 |

Abbreviation: BMI, body mass index; CCI, Charlson comorbidity index; Lap, Laparoscopic; RAS, robotic-assisted Surgery; SD, standard difference
1. 1:1 propensity score matching using the 5-to-1-digit greedy matching algorithm

Supplementary Table 2H. Characteristics of patients underwent lobectomy for lung cancer

|  | **Before Propensity Score Matching** | | | **After Propensity Score Matching**^1^ | | |
| --- | --- | --- | --- | --- | --- | --- |
| **Characteristic** | **VATS N = 6267** | **RAS N = 2060** | **SD** | **VATS N = 1966** | **RAS N = 1966** | **SD** |
| **Age (years), categorical** |  |  |  |  |  |  |
| 18-55 | 697 (11.12) | 202 (9.81) | -0.04 | 185 (9.41) | 193 (9.82) | 0.01 |
| 56-65 | 1695 (27.05) | 505 (24.51) | -0.06 | 482 (24.52) | 489 (24.87) | 0.01 |
| 66+ | 3875 (61.83) | 1353 (65.68) | 0.08 | 1299 (66.07) | 1284 (65.31) | -0.02 |
| **Gender/male** | 2850 (45.48) | 959 (46.55) | 0.02 | 892 (45.37) | 914 (46.49) | 0.02 |
| **Race** |  |  |  |  |  |  |
| White | 5181 (82.67) | 1577 (76.55) | -0.15 | 1575 (80.11) | 1577 (80.21) | 0.00 |
| Black | 533 (8.5) | 130 (6.31) | -0.08 | 118 (6) | 130 (6.61) | 0.03 |
| Other | 553 (8.82) | 353 (17.14) | 0.25 | 273 (13.89) | 259 (13.17) | -0.02 |
| **Primary Payor** |  |  |  |  |  |  |
| Commercial | 1541 (24.59) | 551 (26.75) | 0.05 | 500 (25.43) | 514 (26.14) | 0.02 |
| Medicare | 4100 (65.42) | 1356 (65.83) | 0.01 | 1326 (67.45) | 1302 (66.23) | -0.03 |
| Medicaid | 363 (5.79) | 100 (4.85) | -0.04 | 95 (4.83) | 97 (4.93) | 0.00 |
| Other | 263 (4.2) | 53 (2.57) | -0.09 | 45 (2.29) | 53 (2.7) | 0.03 |
| **BMI≥30** | 659 (10.52) | 259 (12.57) | 0.06 | 219 (11.14) | 244 (12.41) | 0.04 |
| **CCI≥3** | 4613 (73.61) | 1475 (71.6) | -0.04 | 1409 (71.67) | 1415 (71.97) | 0.01 |
| **Inpatient^2^** | 6267 (100) | 2060 (100) | 0.00 | 1966 (100) | 1966 (100) | 0.00 |
| **Provider Region** |  |  |  |  |  |  |
| Midwest | 1095 (17.47) | 254 (12.33) | -0.14 | 237 (12.05) | 254 (12.92) | 0.03 |
| Northeast | 1184 (18.89) | 476 (23.11) | 0.10 | 370 (18.82) | 390 (19.84) | 0.03 |
| South | 2870 (45.8) | 1010 (49.03) | 0.06 | 1061 (53.97) | 1010 (51.37) | -0.05 |
| West | 1118 (17.84) | 320 (15.53) | -0.06 | 298 (15.16) | 312 (15.87) | 0.02 |
| **Urban Hospital** | 5870 (93.67) | 1976 (95.92) | 0.10 | 1881 (95.68) | 1882 (95.73) | 0.00 |
| **Number of Hospital Beds** |  |  |  |  |  |  |
| 0-199 beds | 386 (6.16) | 78 (3.79) | -0.11 | 60 (3.05) | 78 (3.97) | 0.05 |
| 200-499 beds | 2946 (47.01) | 764 (37.09) | -0.20 | 790 (40.18) | 764 (38.86) | -0.03 |
| 500+ beds | 2935 (46.83) | 1218 (59.13) | 0.25 | 1116 (56.77) | 1124 (57.17) | 0.01 |
| **Teaching hospital** | 3590 (57.28) | 1298 (63.01) | 0.12 | 1200 (61.04) | 1204 (61.24) | 0.00 |
| **Physician Volume** |  |  |  |  |  |  |
| Low | 1982 (31.63) | 452 (21.94) | -0.22 | 468 (23.8) | 452 (22.99) | -0.02 |
| Moderate | 1666 (26.58) | 520 (25.24) | -0.03 | 490 (24.92) | 514 (26.14) | 0.03 |
| High | 2619 (41.79) | 1088 (52.82) | 0.22 | 1008 (51.27) | 1000 (50.86) | -0.01 |
| **Physician Specialty** |  |  |  |  |  |  |
| CTS or TS | 5455 (87.04) | 1696 (82.33) | -0.13 | 1764 (89.73) | 1696 (86.27) | -0.11 |
| Other | 812 (12.96) | 364 (17.67) | 0.13 | 202 (10.27) | 270 (13.73) | 0.11 |
| **Procedure year** |  |  |  |  |  |  |
| 2013 | 2051 (32.73) | 705 (34.22) | 0.03 | 665 (33.83) | 668 (33.98) | 0.00 |
| 2014 | 2348 (37.47) | 786 (38.16) | 0.01 | 762 (38.76) | 732 (37.23) | -0.03 |
| 2015 | 1868 (29.81) | 569 (27.62) | -0.05 | 539 (27.42) | 566 (28.79) | 0.03 |

Abbreviation: BMI, body mass index; CCI, Charlson comorbidity index; CTS, cardiothoracic surgery;; RAS, robotic-assisted Surgery; SD, standard difference; TS, thoracic surgery; VATS, video-assisted thoracoscopic surgery.
1. 1:1 propensity score matching using the 5-to-1-digit greedy matching algorithm
2. The characteristic is not used in propensity score matching

Supplementary Table 2I. Characteristics of patients underwent partial nephrectomy for kidney cancer

|  | **Before Propensity Score Matching** | | | **After Propensity Score Matching**^1^ | | |
| --- | --- | --- | --- | --- | --- | --- |
| **Characteristic** | **Lap N = 217** | **RAS N = 4538** | **SD** | **Lap N = 210** | **RAS N = 630** | **SD** |
| **Age (years), categorical** |  |  |  |  |  |  |
| 18-55 | 69 (31.8) | 1561 (34.4) | 0.06 | 67 (31.9) | 221 (35.08) | 0.07 |
| 56-65 | 63 (29.03) | 1373 (30.26) | 0.03 | 62 (29.52) | 168 (26.67) | -0.06 |
| 66+ | 85 (39.17) | 1604 (35.35) | -0.08 | 81 (38.57) | 241 (38.25) | -0.01 |
| **Gender** |  |  |  |  |  |  |
| Male | 122 (56.22) | 2731 (60.18) | 0.08 | 121 (57.62) | 336 (53.33) | -0.09 |
| Female | 95 (43.78) | 1807 (39.82) | -0.08 | 89 (42.38) | 294 (46.67) | 0.09 |
| **Race** |  |  |  |  |  |  |
| White | 158 (72.81) | 3435 (75.69) | 0.07 | 154 (73.33) | 481 (76.35) | 0.07 |
| Black | 14 (6.45) | 424 (9.34) | 0.11 | 14 (6.67) | 30 (4.76) | -0.08 |
| Other | 45 (20.74) | 679 (14.96) | -0.15 | 42 (20) | 119 (18.89) | -0.03 |
| **Primary Payor** |  |  |  |  |  |  |
| Commercial | 108 (49.77) | 2163 (47.66) | -0.04 | 106 (50.48) | 337 (53.49) | 0.06 |
| Medicare | 82 (37.79) | 1795 (39.55) | 0.04 | 79 (37.62) | 229 (36.35) | -0.03 |
| Medicaid | 19 (8.76) | 327 (7.21) | -0.06 | 17 (8.1) | 41 (6.51) | -0.06 |
| Other | 8 (3.69) | 253 (5.58) | 0.09 | 8 (3.81) | 23 (3.65) | -0.01 |
| **BMI≥30** | 46 (21.2) | 931 (20.52) | -0.02 | 45 (21.43) | 130 (20.63) | -0.02 |
| **CCI≥3** | 78 (35.94) | 2028 (44.69) | 0.18 | 77 (36.67) | 216 (34.29) | -0.05 |
| **Inpatient**^2^ | 217 (100) | 4538 (100) | 0.00 | 210 (100) | 630 (100) | 0.00 |
| **Provider Region** |  |  |  |  |  |  |
| Midwest | 22 (10.14) | 947 (20.87) | 0.30 | 22 (10.48) | 67 (10.63) | 0.01 |
| Northeast | 130 (59.91) | 909 (20.03) | -0.89 | 123 (58.57) | 352 (55.87) | -0.05 |
| South | 50 (23.04) | 1862 (41.03) | 0.39 | 50 (23.81) | 181 (28.73) | 0.11 |
| West | 15 (6.91) | 820 (18.07) | 0.34 | 15 (7.14) | 30 (4.76) | -0.10 |
| **Urban Hospital** | 207 (95.39) | 4301 (94.78) | -0.03 | 201 (95.71) | 603 (95.71) | 0.00 |
| **Number of Hospital Beds** |  |  |  |  |  |  |
| 0-199 beds | 15 (6.91) | 408 (8.99) | 0.08 | 15 (7.14) | 45 (7.14) | 0.00 |
| 200-499 beds | 44 (20.28) | 1961 (43.21) | 0.51 | 44 (20.95) | 137 (21.75) | 0.02 |
| 500+ beds | 158 (72.81) | 2169 (47.8) | -0.53 | 151 (71.9) | 448 (71.11) | -0.02 |
| **Teaching hospital** | 158 (72.81) | 2449 (53.97) | -0.40 | 151 (71.9) | 451 (71.59) | -0.01 |
| **Physician Volume** |  |  |  |  |  |  |
| Low | 100 (46.08) | 1210 (26.66) | -0.41 | 93 (44.29) | 280 (44.44) | 0.00 |
| Moderate | 38 (17.51) | 1350 (29.75) | 0.29 | 38 (18.1) | 98 (15.56) | -0.07 |
| High | 79 (36.41) | 1978 (43.59) | 0.15 | 79 (37.62) | 252 (40) | 0.05 |
| **Procedure year** |  |  |  |  |  |  |
| 2013 | 78 (35.94) | 1412 (31.12) | -0.10 | 76 (36.19) | 238 (37.78) | 0.03 |
| 2014 | 98 (45.16) | 1750 (38.56) | -0.13 | 93 (44.29) | 282 (44.76) | 0.01 |
| 2015 | 41 (18.89) | 1376 (30.32) | 0.27 | 41 (19.52) | 110 (17.46) | -0.05 |

Abbreviation: BMI, body mass index; CCI, Charlson comorbidity index; Lap, Laparoscopic; RAS, robotic-assisted Surgery; SD, standard difference
1. 1:3 propensity score matching using the 8-to-1-digit matching algorithm.
2. characteristic is not used in propensity score matching
